# Strategies associated with improved healthiness of consumer purchasing in supermarket interventions: a systematic overview of reviews and evaluation of primary articles

**DOI:** 10.3389/fpubh.2024.1334324

**Published:** 2024-06-25

**Authors:** Paige G. Brooker, Caitlin A. Howlett, Emily Brindal, Gilly A. Hendrie

**Affiliations:** Health and Biosecurity, Commonwealth Scientific and Industrial Research Organisation (CSIRO), Adelaide, SA, Australia

**Keywords:** supermarket, retail food environment, public health, health promotion, review

## Abstract

**Background:**

Growing evidence suggests that it is possible to change the retail food environment to enable healthier choices via in-store interventions. It has been difficult to draw clear conclusions as to which interventions are most effective in positively influencing consumer purchasing behaviour given the significant heterogeneity within the food retail research literature. The aim of this study was to (1) summarise current high-quality systematic, scoping, and/or narrative reviews (Part I: overview of reviews); and (2) synthesise high-quality original research, to understand the range, types and effectiveness of strategies implemented in food retail settings (Part II: evaluation of primary studies).

**Methods:**

To identify reviews describing the effects of intervention strategies aiming to improve the healthiness of consumer purchasing in supermarkets, a systematic search across seven electronic databases was completed in April 2023. The methodological quality of reviews was assessed using the risk of bias in systematic reviews for systematic and scoping reviews, and the Scale for the Assessment of Narrative Review Articles for narrative reviews. High-quality reviews were further inspected and synthesised narratively (Part I). Next, to understand strategies associated with improved healthiness of consumer purchasing high-quality, primary articles from high-quality reviews identified in Part I were retrieved, and the strategies implemented within these interventions were summarised (Part II).

**Results:**

Thirty-eight reviews met the inclusion criteria for Part I; two-thirds (*n* = 25, 66%) were rated as high-quality (66%). These reviews indicated that pricing strategies had the greatest proportion of reported positive or promising effects on outcomes (*n* = 8 of 11 reviews, 73%). Twenty reviews met the inclusion criteria for Part II and the 771 primary articles from these reviews were screened with 23 high-quality primary articles included in analysis. Findings indicated that promotional strategies in combination with another strategy appeared to be most successful among regular shoppers (the general population), whereas pricing was most successful in low socio-economic status and rural sub-groups.

**Conclusion:**

Promotion, pricing and prompting were the most commonly tested strategies across the overview of reviews and review of primary articles. Promotion, in combination with other strategies, and pricing appear to be most promising, but the effectiveness of pricing strategies may vary by sub-groups of the population. How pricing and promotion in combination with other strategies can be implemented responsibly and sustainably to change purchase habits towards healthier items should be explored further.

**Systematic Review registration:**

OSF, https://osf.io/jyg73/.

## 1 Introduction

Poor dietary intake, characterised by lower intakes of whole grains, fruits, nuts and seeds, higher intakes of red meat and sugar-sweetened beverages, is a leading driver of morbidity and premature mortality, globally (1). Dietary intake is influenced by a range of individual, social, environmental and system level factors (2–4). Supermarkets, as one actor in the food system, influence population diets through the creation of retail environments that shape food purchases, and ultimately consumption, through manipulating layout, availability, price, and promotion (5, 6).

In developed countries, households purchase nearly all their food within a retail setting (7). In Australia, two-thirds of all food purchased is from supermarkets (8), with similar figures in the US (9) and United Kingdom (10). Previous research has shown that individuals living in areas with greater availability of supermarkets have a lower body mass index (11). Living near healthier food stores is also associated with better diet quality (12). Supermarket purchase behaviour can be habitual, but is not often planned in detail (13), meaning consumers’ purchasing behaviour could be shifted by changing the in-store retail food environment to be more health enabling (14).

Currently, supermarkets actively attempt to influence purchasing through techniques typically grouped into the ‘four Ps of marketing’—product, price, placement, and promotion (5, 6). Published literature provides examples of enabling strategies within each ‘P’ such as reducing/replacing unhealthy foods (product); using price reductions to increase acceptability of unfamiliar healthier foods (price); placing multiple healthy checkout aisles in stores to shift the healthy/unhealthy balance (placement); and highlighting healthy options by displays, labels and samples to taste (promotion) (6). The effectiveness of interventions using such strategies is mixed (15–18). It has been difficult to draw clear conclusions as to which of the four P strategies, or combination thereof, is most effective in positively influencing consumer purchasing behaviour given the significant heterogeneity within the food retail research literature in terms of the effectiveness of such interventions, as well as the types of populations and settings included.

Several reviews (6, 19–26), and updates of reviews (27, 28) investigating the effectiveness of interventions on improving the healthfulness of the retail food environment have been published over the past two decades. These have been undertaken across a broad range of food retail outlets including convenience stores, vending machines, quick-service restaurants, and school or workplace cafeterias, and few have focused exclusively on supermarket settings. This is important, since these other settings have attributes distinct from supermarkets, and account for a much lower portion of individuals’ food and beverage purchases (13). Existing reviews also include studies conducted in mock (simulated) supermarkets, or laboratory settings, which is less ecologically valid and likely less reflective of natural behaviour (29). Given the central role of supermarkets in shaping population diets, the supermarket food environment should be given focussed consideration as an avenue to improve eating habits.

The objective of this study was to review the available evidence on the effectiveness of real-world supermarket-based interventions on the healthiness of consumer purchases and consumption. Given the existing high volume of literature on this topic, an overview of reviews was considered appropriate to synthesise existing findings and provide a rapid synthesis of high-quality evidence. Overviews of reviews (also known as ‘umbrella’ reviews) are common practice and integrate the findings of multiple previously published reviews, allowing rapid assessment of the evidence base on a topic area (30, 31). Therefore, the first aim was to summarise the current body of high-quality evidence obtained from systematic, scoping, and narrative reviews (Part I). The second aim was to interrogate this high-quality secondary research to better understand the range and effectiveness of strategies evaluated in food retail settings (Part II).

## 2 Methods

### 2.1 Overview

**Figure 1:**
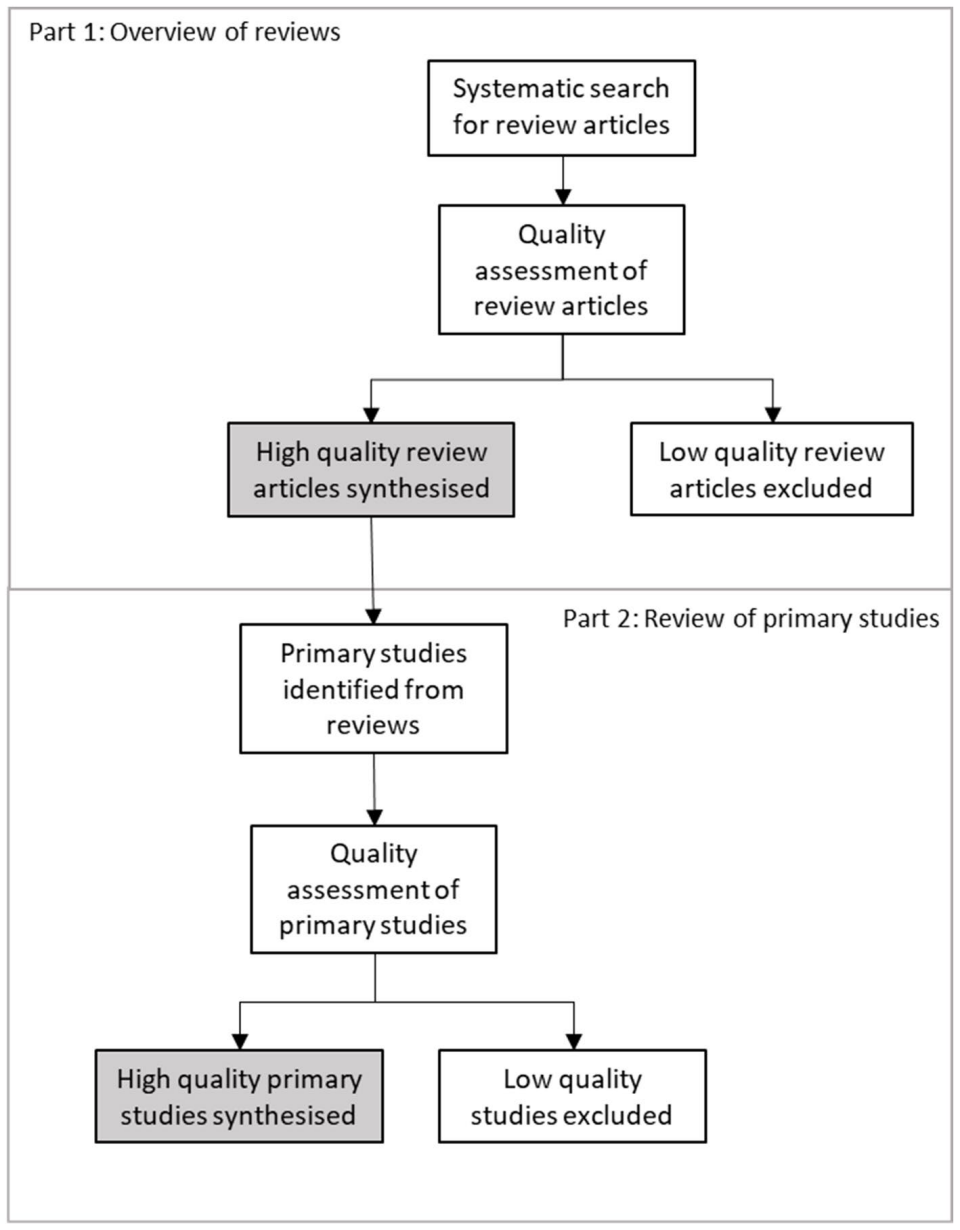
Summary of the methodological approach to sourcing and including articles in the two parts of this review: Part 1, Overview of reviews; and Part 2, Review of primary studies.

The first stage of this research was to use a systematic process to synthesise current evidence through an umbrella review (Part I). To understand what strategies are most likely to be effective in changing purchase patterns of consumers in supermarkets, we then undertook a comprehensive review of high-quality primary research studies, identified from high-quality review articles (Part II). This synthesis aimed to provide a deeper understanding of the strategies implemented within supermarket-based studies, and key learnings about their relative success and failure in improving the healthiness of consumer purchasing. Figure 1 provides an overview of the two parts of this review.

### 2.2 Information sources and search strategy

This review was guided by recommendations for the conduct of overviews of reviews from the Cochrane Handbook (32) and findings of reviews are reported based on suggestions in the Preferred Reporting Items for Overviews of Reviews (PRIOR; (33)) guidelines. The study objective, search strategy, selection criteria and synthesis plan were specified *a priori* (see study protocol in Appendix A) and uploaded to Open Science Framework,^1^ retrospectively.

A literature search was conducted in March–April 2023 across seven databases: PubMed, Web of Science (core collection), Scopus, ProQuest, EconLit, Cochrane Central and Google Scholar (retrieving the first 200 results). The search strategy was developed by the authors in conjunction with an expert librarian using a modified PI(E)COCS framework (Population, Intervention/Exposure, Comparison, Outcome, Context, and Study Design; Table 1) (34). Briefly, reviews that reported on the effectiveness of strategies implemented in supermarkets or grocery stores that aimed to improve the healthiness of food and/or beverages purchased or consumed by consumers were included. Accreditation schemes are a type of promotion and a potentially important lever for influencing consumer behaviour but occur at a system level. Furthermore, in light of a recent review focused specifically on the effectiveness of outlet-level healthy food and beverage accreditation schemes (35), these strategies were considered beyond the scope of this review.

**Table 1:** PI(E)COCS criteria.

Criteria	Inclusion	Exclusion
Population	Supermarket shoppers	Children or persons who do not make food purchases independently
Intervention/Exposure	Interventions that aimed to improve the healthiness of food purchased by consumers by altering factors such as product, price, promotion, placement	Interventions that focused on new stores Interventions related to government taxation or money subsides Interventions operating at the manufacturer, rather than retailer, level (e.g., product reformulation, food labelling) Accreditation schemes
Comparator	No restrictions	
Outcome	Food/beverage purchasing information – can be objective (e.g., sales data, shopper receipts) or self-reported (purchase behaviour); or Dietary consumption (e.g., food recall); or Health outcomes (e.g., weight change)	Alcoholic drink sales/consumption Hypothetical choice/purchase intentions Economic evaluation Shelf-space or availability of products
Context	Real-world, physical or online supermarket or grocery stores* where there is an exchange of money for food and beverage products to be consumed elsewhere	Other food retail environments such as farmers markets, food pantries, convenience stores, corner stores, cafeterias/restaurants, vending machines, school/workplace canteens, sporting venues^#^ Simulation or laboratory studies
Study design (Part I)	Review articles (scoping, systematic, literature, umbrella)	Primary research articles
Study design (Part II)	Primary research articles, no restrictions were placed on study design	Reviews, conference abstracts, study protocols

*The retail literature does not use the terms ‘grocery store’ and ‘supermarket’ interchangeably, so we accepted each term as presented by the author (s) (27); ^#^Reviews that included mixed settings were eligible if at least one of the settings contained supermarket or grocery stores (e.g., reviews of supermarkets and convenience stores).

A combination of MeSH (medical subject headings) terms and free-text keywords were used to search for relevant interventions (e.g., ‘product availability’, ‘choice architecture’, ‘price’, or ‘promotion’) and the outcomes of interest (e.g., ‘healthy eating’, ‘diet quality’, ‘sales data’ or ‘customer satisfaction’). The detailed search strategy is available in Appendix B. The reference lists of included reviews and relevant review articles were searched to capture any citations missed by electronic searches (‘backward search’). Search parameters were limited to review articles published in the English language (the native language of the authors). No date restrictions were applied; the search included review articles published from database inception through to 4 April 2023.

### 2.3 Review selection

Citations and abstracts of all retrieved records were imported to EndNote (X9) (36). Duplicate records were identified and removed, and the remaining citations imported to Covidence (37). Records were assessed for eligibility against the PI(E)COCS criteria (Table 1), initially screened based on their title and abstract; any records that were potentially eligible were advanced to full-text review. Study selection was performed by two reviewers (PB and CH), independently. Conflicts in the selection process were resolved by discussion until a consensus was reached.

### 2.4 Quality appraisal of reviews

The search retrieved all review types, including systematic, scoping, and narrative reviews. To identify high-quality reviews, assessments were conducted using published quality appraisal tools specific to each review type. Currently, there are no internationally established standards for critically appraising or determining risk of bias in scoping reviews (38), therefore, the Risk of Bias In Systematic Reviews (ROBIS) (39) was used to appraise both scoping and systematic reviews. To assess the quality of narrative reviews, the Scale for the Assessment of Narrative Review Articles (SANRA) was used (40).

As per instructions, the ROBIS tool was completed in two phases: (i) identify bias with the review process, and (ii) judge the overall risk of bias in the review. In phase one, the risk of bias was assessed across four domains: study eligibility criteria; identification and selection of studies; data collection and study appraisal; and synthesis and findings. The level of risk of bias associated within any of the domains in phase 1 was graded to categorise the overall risk of bias (referred to as study quality hereafter) as *low*, *high*, or *unclear* (phase 2).

The SANRA tool assesses the quality of narrative reviews across six domains: explanation of the review’s importance; statement of the aims; description of the literature search; referencing; scientific reasoning; and presentation of relevant and appropriate endpoint data. Each domain is scored out of 2, and summed to give a total score out of 12; a score of 4 or below indicates very poor quality (40). The SANRA tool does not provide a cut-off score to indicate whether a review can be considered ‘high-quality’. For this study, two investigators (PB and CH) agreed on ‘critical’ domains and a subsequent scoring system to assess the overall quality of reviews. Articles were considered high-quality if they scored two (maximum score) for each critical domain and did not score poorly (zero) in more than one other domain (Appendix C). The quality assessment was performed in duplicate by two independent reviewers (PB and CH). Disagreements were resolved by consensus between the two reviewers.

### 2.5 Primary article selection

Where a quality assessment was completed within high-quality reviews, primary articles deemed to be high-quality (based on criteria established by the original review authors) were retrieved. Where reviews used a risk of bias tool that do not provide an overall quality rating of primary articles, two authors (PB and CH) decided on critical domains from each quality assessment tool and used the review authors scoring on these domains to categorise primary articles as high-quality, or not (Appendix D). There is no standard approach to deal with overlap in primary articles across reviews (41). Therefore, when primary articles were included in more than one high-quality review, the quality rating from the most recently published and highest quality review was chosen; an approach suggested by Lunny and colleagues (42, 43). The retrieved primary research articles were examined for eligibility against PI(E)COCS criteria (Table 1).

### 2.6 Data extraction and synthesis

A standardised data extraction template was created in Microsoft Excel^®^ (Version 2022), and used to collect the following information from the included reviews and primary articles: (i) Publication Details: first author’s family name, year of publication; (ii) Review/Study Characteristics: primary objective, inclusion criteria and search restrictions (reviews only), study design (primary studies only), and retail setting(s); (iii) Intervention Characteristics: details regarding intervention and control treatments; (iv) Outcomes: methods used to assess outcomes, and outcome results; and (v) Study Conclusions: main conclusions as reported by authors. Data from each review and primary study were extracted by one author (PB or CH) and checked by a second investigator (CH or PB). Data were synthesised narratively. The type of in-store intervention described in articles was categorised according to the framework by Kraak et al. (44), and adapted for use in grocery store settings by Slapø et al. (13) (Table 2). The framework was adapted further to include ‘*product availability’*, and ‘*combined’* strategies. Outcome effects were coded using ratings proposed by Chan et al. (46). Outcome effect ratings included: (i) ‘positive’, where there was a positive effect on the primary outcomes as intended; (ii) ‘promising’, positive effect potentially with change in power, dose, exposure, or analysis; (iii) ‘mixed due to intervention’, mixed outcomes due to different treatment arms having different effects; (iv) ‘mixed due to outcomes’, positive findings for some outcomes, negative, or no effect for other outcomes; (v) ‘no effect’, no effect on any outcome; (vi) ‘negative’, effect in opposite direction as intended; or (vii) ‘unclear’, inappropriate analysis or insufficient evidence to support outcome. Where a review or primary study reported separate syntheses of the effects of different intervention strategies, information describing the effects of each synthesis was extracted. If multiple time points were reported, only the end of the intervention point and final follow-up were used. Where information was missing from the published manuscripts, authors were contacted twice over a two-week period to provide the additional information.

**Table 2:** Strategies to promote healthy food and beverage environments in grocery stores.

Strategy	Description
Portioning	Reduce and/or standardise the portion size of food and beverage products that meet recommended nutrient targets to influence customers’ expectations about single servings and appropriate portions to support healthy dietary guidelines
Place	Changing the internal setting (e.g., lighting, smell, music and branding of stores) that impact the ambience or atmospherics to highlight healthy food and beverage products.
Proximity	Placing healthier products at eye level or physically closer to customers at point-of-choice and point-of-purchase (e.g., placing healthier options at the entry or exit of store and giving healthy options better placement in the shelf).
Promotion	Use of marketing practices inside store that support healthier diets (i.e., products samples, taste-testing, in-store demonstrations, inside store audio public service announcements and education sessions inside store to promote healthy products).
Healthy Default Picks	Use of environmental cues that are convenient, accepted and expected to socially normalise healthy defaults choices (e.g., introducing swaps that offer customers the opportunity to replace their usual food with healthier alternatives).
Pricing	Use of pricing strategies to increase sales of products that meet recommend nutrient targets to support healthy dietary guidelines (e.g., changes in price per unit, coupons and cash-back).
Prompting	Use of information on products to help customers make healthier choices at point-of choice and point-of-purchase (e.g., guiding star labelling system, nutrition labels and traffic-light labels).
Profile	Change in the product’s nutritional profile, quality, smell, taste, texture, flavour of food or beverage products that make meeting nutritional targets according to dietary guidelines.
Product Availability	Increasing, decreasing, or changing the range or number of product options available to customers [as defined by Hollands et al. (45)].
Combined*	Reviews that included a high-level narrative synthesis of either (i) multiple single-component strategies or (ii) both single-and multi-component strategies that were synthesised together, and thus the results could not be attributed to a particular strategy.

Adapted from “Efficiency of In-Store Interventions to Impact Customers to Purchase Healthier Food and Beverage Products in Real-Life Grocery Stores: A Systematic Review and Meta-Analysis,” by H. Slapø, 2021, foods, 10 (922). *applicable for Part I only; Reviews that only synthesised studies examining the effects of ‘Portioning’ and ‘Profile’ strategies were not included in this review.

### 2.7 Deviations from the pre-registered study protocol

Some changes to the methods outlined in the pre-registered protocol were necessary. Overviews of reviews were planned for inclusion to capture all available (consolidated) evidence in the research area. Following execution of the search strategy and study screening, umbrella reviews were excluded from further analysis. We did, however, examine the reference lists of eligible umbrella reviews (Gupta et al. (47), Roberts et al. (48) and Wolfenden et al. (49)) to cross-check for the inclusion of relevant review articles.

Reviews that focused on interventions related to food labelling or taxation/money subsidies were pre-planned exclusion criteria. After examining the search results, it became apparent that these broad terms encompassed strategies deemed eligible for inclusion in the review. For example, ‘food labelling’ may include promotion of products via shelf-tags (included), not just front-of-pack labelling (excluded), and taxation/money subsidies may include pricing discounts in-store (included), not just government taxation initiatives such as ‘sugar tax’ (excluded).

## 3 Results

### 3.1 Part I—overview of reviews

**Figure 2:**
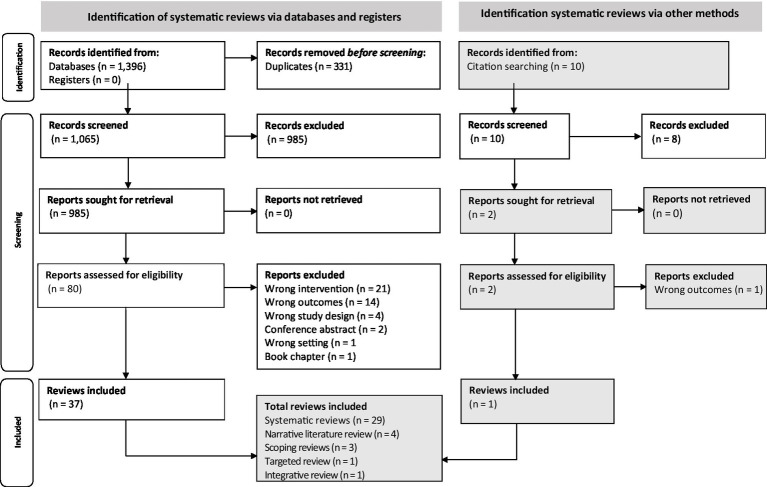
Preferred reporting items for overviews of reviews (PRIOR) flowchart for study selection.

The literature search resulted in a total of 1,406 records. After the removal of duplicates (*n* = 331), a total of 1,075 abstracts were initially screened by title and abstract. Eighty-two abstracts were eligible for full-text review. A total of 38 review articles met the eligibility criteria and were included in this overview of reviews (Figure 2).

#### 3.1.1 Quality assessment of included reviews

The quality of the review articles was assessed using the ROBIS or SANRA tools. Appendix C shows the full quality appraisal, including how the reviews scored on each domain. Twenty-five (66%) were rated as high-quality (low risk of bias), and the remainder were rated as low-quality (high risk of bias; *n* = 4), or the quality was unclear (*n* = 9).

The focus of this overview of reviews was on high-quality reviews. Therefore, results will be presented only for high-quality reviews. Characteristics of low-quality reviews, or those where the quality was unclear, can be found in Appendix E.

#### 3.1.2 Review characteristics

The characteristics of the 25 high-quality reviews are presented in Table 3. Reviews were published between 2014 (19) and 2023 (60). Most were systematic reviews, and included between eight (62) and 107 (52) primary articles. About a third of the reviews (*n* = 9 of 25, 36%) also searched grey literature (45, 52–55, 60, 61, 67, 68).

**Table 3:** Characteristics of high-quality reviews included in the overview of reviews.

Reference (author, year)	Review type	Review eligibility criteria			Search strategy		No. of included primary studies
		Research design	Population and setting	Intervention strategy	No. of databases	Search period	
Adam & Jensen, 2016 (14)^^^	Systematic review	Intervention studies	Population: NR Setting: Physical retail food stores (grocery stores, supermarkets, and convenience stores)	Affordability (price), information and access/availability	3	2003 to 2015 (inclusive)	42
Afshin et al., 2017 (50)^^^	Systematic review + meta-analysis	RCTs and non-RCTs; prospective observational	Population: Adults and children Setting: Supermarkets, restaurants, schools, workplace, fast food, cafeterias	Change in the price of foods or beverages (i.e., taxation, subsides, or other factors)	7	NR (1992 to 2014)^§^	30
Alston et al., 2020 (51)^^^	Systematic review	NR	Population: NR Setting: Food retail environment in a rural, non-urban, remote, regional, or non-metropolitan area in any country	Food retail environment initiatives	3	1 Jan 2000 to 31 May 2020	21
Blake et al., 2019 (52)^^^	Systematic scoping review	NR	Population: NR Setting: Grocery and convenience stores, supermarkets, fresh food markets, bakeries, and specialty food stores; Restaurants and Other Eating Places including cafeterias and cafes; vending machine merchandisers, sale of products related to food and beverages	4Ps (product, place, price, promotion) or any combination of these	8 + grey literature searched	Jan 1997 to Jul 2017	107
Cameron et al., 2016 (53)^^^	Systematic review	Intervention studies (investigator led or natural experiment)	Population: NR Setting: Supermarkets, grocery stores and online stores	Changed the in-store environment to influence consumer nutrition/diet (i.e., product, promotion, or place)	5 + grey literature searched	No date limits	50
Crockett et al., 2018 (54)^^^	Systematic review (Cochrane)	RCTs, Q-RCTs, cluster-randomised studies, ITS and CBA	Population: Adults or children Setting: Any retail outlet (grocery stores, food stores, vending machines, cafeterias, and both fast and non-fast-food restaurants); real-world or laboratory	Nutritional labelling of a food or non-alcoholic drink product	13 + grey literature searched	Database inception to 26 Apr 2017.	28
Fergus et al., 2021 (55)^^^	Systematic review	NR	Population: NR Setting: Rural and urban low-income retail food stores	Retail nutrition intervention besides interventions offering solely financial incentives	5 + grey literature searched	Oct 2010 to Oct 2019	46
Gittelsohn et al., 2017 (56)^^^	Systematic review	Experimental studies (RCTs, quasi-experimental, natural experiments)	Population & setting: Population studies of people or stores in middle-income and high-income countries (real-world)	Pricing incentive and disincentive strategies (alone or combined with health behaviour interventions or as part of multi-level strategies)	6	Jan 2000 to Dec 2016	30
Golding et al., 2022 (57)^^^	Systematic review	RCTs and non-RCTs	Population: In-store shoppers Setting: Physical supermarkets	Any intervention aimed at influencing shoppers’ food and non-alcoholic drink purchasing behaviour	11	Database inception to Jan/Feb 2017	46
Harbers et al., 2020 (58)^^^	Systematic review	NR	Population: Adults Setting: Real-life food purchasing environments where food or meal purchases can be made on a regular basis	Nudging (i.e., availability, position, functionality, presentation, size, information)	3	Database inception to 31 Jan 2018	75
Hartmann-Boyce et al., 2018 (59)^^^	Systematic review	RCTs	Population: No restrictions Setting: Physical, online, or simulated grocery store	Interventions designed to change the purchase of any foods, non-alcoholic drinks, nutrients, energy, or products belonging to a defined dietary pattern or with defined dietary scores	13	NR (Search performed 2 Jun 2017)	35
Hodges et al., 2023 (60)^^^	Systematic review	Intervention, observational or qualitative studies	Population: Consumers Setting: Online grocery shopping platform (real-world or laboratory)	Retail marketing strategies (product suggestions, promotions, price etc.)	6 + grey literature searched	1 Jan 2015 to May/Jun 2022	18
Hollands et al., 2019 (46)^^^	Systematic review (Cochrane)	RCTs or cluster-RCTs with between-participants (parallel group) or within-participants (cross-over) designs	Population: Adults and children Setting: Restaurants, workplaces, schools, homes, bars, pubs, supermarkets, or shops (real-world or laboratory)	Availability and proximity interventions	8 + grey literature searched	Inception Database inception to 23 Jul 2018	24
Karpyn et al., 2020 (28)^^^	Systematic review	Intervention, pilot, or experimental studies	Population: Customers Setting: Food retail environment (i.e., supermarket, grocery store, corner store, bodega, retail environment)	4Ps (product, place, price, promotion); either single or multi-component interventions	9	2010 to 2019	64
Liberato et al., 2014 (19)^^^	Systematic review	RCTs, CBA studies or ITS designs and analyses.	Population: General population and/or organisations Setting: Supermarket, grocery store and/or vending machine	Nutrition interventions at the point-of-sale aiming to (i) impact availability, affordability and/or ability to choose healthier foods and drinks, (ii) to influence food and drink purchases (including, infrastructure or monetary incentives as well as marketing strategies including promotion and placement strategies), or (iii) any combination of these	3	No date limits	32
Mah et al., 2019 (27)^^^	Systematic review	Quantitative, qualitative, or mixed methods	Population: General population Setting: Real-world grocery stores, supermarkets, convenience stores, and gas stations	Altering the availability or mix of retailers in a geographic area (community food environment) or the 4Ps (product, pricing, placement, or promotion) in-store	3	Database inception to Nov 2018	86
Nikniaz et al., 2020 (61)^^^	Systematic review	RCTs or quasi-experimental studies	Population: All population groups Setting: NR	Community-based interventions aimed at increasing dairy/calcium consumption	6 + grey literature searched	2000 to 2019	25
Nikolaus et al., 2016 (62)^^^	Systematic review	No restrictions	Population: No restrictions Setting: Grocery stores, supermarkets	Supermarket/grocery store tours	2	Jan 1984 to Apr 2015	8
Shangguan et al., 2019 (63)^^^	Systematic review + meta-analysis	RCTs and non-RCTs	Population: NR Setting: Restaurants, supermarkets, grocery stores, cafeterias, food retail/self-service establishments, and vending machines	Food labelling	10	Database inception to 28 Feb 2014	60
Shaw et al., 2020 (64)^^^	Systematic review	Intervention and observational studies	Population: Individuals >18 years Setting: Supermarkets, convenience stores	Positioning or availability of food/beverage items	9	Jan 2005 to Feb 2019	38
Slapø et al., 2021 (13)^^^	Systematic review + meta-analysis	RCTs, CBA or ITS	Population: No restrictions Setting: Grocery stores (real physical or real online)	Choice architecture interventions (portioning, place, proximity, promotion, healthy default picks, pricing, prompting, profile)	6	NR (Search performed on 24 Apr 2020)	36
Valencic et al., 2022 (65)^^^	Scoping review	NR	Population: Adults Setting: Online grocery stores or supermarkets	Interventions using a digital nudging approach (manipulated the user-interface)	8	Database inception to Feb 2022	15
Volkova et al. 2015 (66)^*^	Narrative literature review	Experimental and real-life designs	Population: NR Setting: Retail settings	Nutrition labels and point-of-purchase information	NR	2011 to 2014	30
von Philipsborn et al., 2019 (67)^^^	Systematic review (Cochrane)	RCTs, non-RCTs, CBA, RMS, or ITS	Population: Any (adults, adolescents, or children) Setting: Real-world settings	Environmental interventions (i.e., labelling, nutrition standards, economic tools, advertisement regulation, whole food supply, retail and food service, action across sectors)	11 + grey literature searched	Database inception to 24 Jan 2018	58
Wyse et al., 2021 (68)^^^	Systematic review + meta-analysis	RCTs, cluster-RCTs, stepped-wedge RCTs, factorial RCTs, multiple baseline RCTs, randomised controlled crossover trials, quasi-randomised controlled trials, or CCTs	Population: Generally healthy participants Setting: Online supermarkets and grocery stores, online restaurants, cafes, and canteens; and online food and meal delivery services	Dietary interventions delivered via online food ordering systems	8 + grey literature searched	Database inception to 1 Oct 2020	11

^^^indicates study quality was assessed using the ROBIS tool; ^*^indicates study quality was assessed using the SANRA tool; (44); ^§^extrapolated from results. CBA, controlled before and after; CCT, clinical controlled trial; ITS, interrupted time series; NR, not reported; Q-RCT, quasi-randomised controlled trial; RCT, randomised controlled trial; RMS, repeated measures studies.

Of the 25 reviews, most (*n* = 19, 76%) included a range of food retail settings, such as supermarkets, convenience stores, cafeterias, farmers markets, vending machines and canteens. Only six reviews (24%) focused exclusively on primary studies conducted in supermarkets and/or grocery stores; two of which were conducted solely in physical brick and mortar supermarkets (57, 62) while the other four were conducted in a combination of real-world physical stores, real-world online stores or simulated supermarket environments (13, 53, 60, 65) (Appendix F).

Reviews mostly focused on ‘regular shoppers’ as the population of interest (*n* = 17 of 25, 68%); one focused on people or stores from middle-income and high-income countries (56). Seven reviews did not specifically state the eligible population(s) as part of their PICO framework (14, 51–53, 55, 63, 66).

The most assessed strategies in reviews were pricing (*n* = 9 of 25, 36%), promotion (*n* = 8, 32%) and availability (*n* = 8, 32%). Other strategies less commonly evaluated in reviews included proximity (*n* = 7, 28%), prompting (*n* = 6, 24%), place (*n* = 2, 8%) and healthy default picks (*n* = 1, 4%). Seven of the reviews (28%) focused on a single intervention strategy—four solely on prompting strategies (54, 63, 66, 67); two solely on pricing strategies (50, 56); and one on promotion strategies (62). Almost half the reviews (*n* = 11 of 25, 44%) evaluated ‘combined’ intervention strategies.

Reviews needed to report outcomes related to purchase/sales, consumption, or health outcomes to be included. Most reviews (*n* = 21 of 25, 84%) (13, 14, 19, 27, 28, 45, 50, 51, 53–60, 62–66, 68) assessed the effects of intervention strategies on objective (e.g., sales data, customer receipts) or subjective (e.g., survey self-reported purchases, intent to purchase, or direct in-store observation) purchase-related outcomes (Appendix F). Sixteen reviews (64%) (19, 27, 28, 45, 50, 51, 53–56, 58, 61–64, 67) assessed the effects of intervention strategies on consumption as the primary outcome, and three reviews considered consumption as a secondary outcome (14, 45, 59). Seven reviews (19, 27, 50, 53, 63, 64, 67) assessed the effects of intervention strategies on health outcomes (e.g., body weight/composition, BMI, metabolic risk factors or clinical endpoints); and two included this as a secondary outcome (19, 50).

In addition to outcomes forming inclusion criteria for the current study, reviews reported outcomes such as business-related outcomes (e.g., retailer/customer perceptions, commercial viability, community outcomes, storeowner attitudes), industry responses (e.g., changes in formulations or availabilities of products) (52, 55, 56, 63, 68), or consumer knowledge, beliefs, preferences or intentions, nutrient content of baskets, or cost-effectiveness (health-care savings) (19, 54, 58, 60, 62, 65, 67, 68).

#### 3.1.3 Review findings

**Figure 3:**
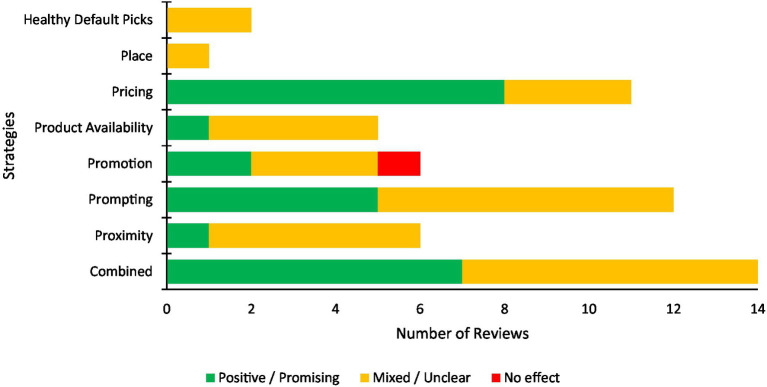
Summary of the findings of the included reviews investigating the effectiveness of changing consumer purchases by strategy type.

Prompting was the most common single component strategy across the 25 included reviews. Of the 12 reviews that evaluated prompting as a strategy, five (42%) reported positive/promising effects on the outcomes measured, while seven (58%) reported mixed/unclear effects. As a single component strategy, pricing was most successful with the greatest proportion of reviews reporting positive or promising effects on outcomes (*n* = 8 of 11 reviews, 73%). A total of 14 reviews reported combined strategies, half of which reported positive/promising effects. Pricing plus another strategy was common among the reported multi-component strategies. A summary of the review findings by strategy type are illustrated in Figure 3.

#### 3.1.4 Quality assessment of primary articles included in reviews

Three of the 25 high-quality reviews (12%) did not appraise the quality/bias of the primary articles the included in their review (27, 65, 66). The appraisal tools used to assess primary articles varied among the remaining 22 high-quality reviews, but two common tools were the Cochrane Collaboration Risk of Bias tool or an adapted version (13, 14, 28, 45, 54, 59, 67, 68) (*n* = 8 of 22, 36%), and the Effective Public Health Practice Project Quality Assessment Tool for Quantitative Studies (*n* = 5 of 22, 23%) (19, 51, 53, 55, 58) (Appendix F).

### 3.2 Part II—review of primary research articles

The scope and objectives of the review articles varied, and as a result the intervention types and settings of the primary studies within the reviews also varied greatly. Given this heterogeneity, it was difficult to conduct a quantitative synthesis from the reviews on strategies, and their effectiveness when implemented in supermarkets or grocery stores. Therefore, to achieve this level of granularity, high-quality primary studies conducted in supermarkets or grocery stores were identified from the 25 high-quality review articles.

**Figure 4:**
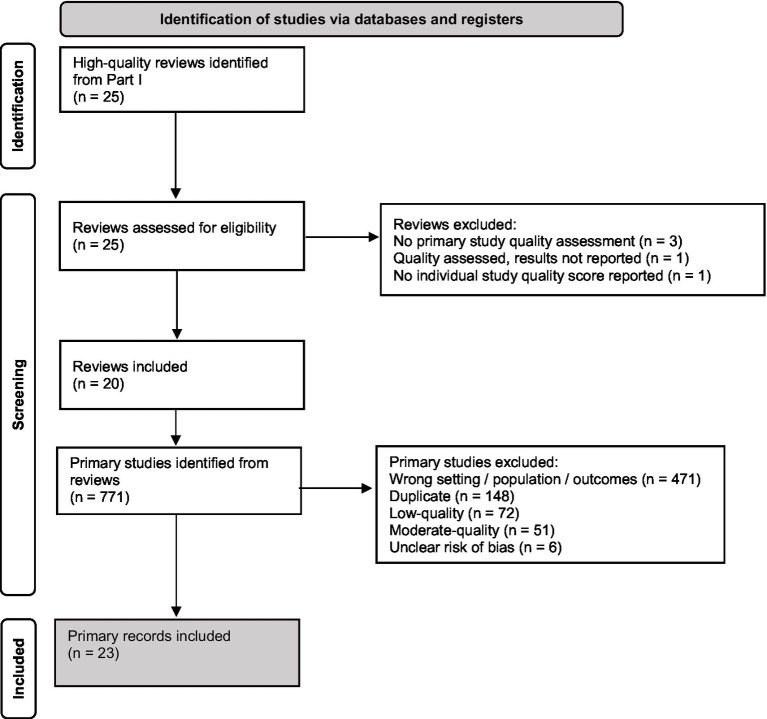
Preferred reporting items for systematic reviews (PRISMA) flow diagram for study selection.

Five high-quality reviews were excluded from further inspection because they did not include a quality assessment of the primary research (*n* = 3) (27, 65, 66); did not report the results of their quality appraisal of primary research articles in text (and did provide the requested material when contacted; *n* = 1) (28); or presented aggregated results, so the quality of individual articles could not be evaluated (*n* = 1) (56). Finally, primary research articles from 20 high-quality reviews were sourced and screened (Figure 4).

Seven-hundred and seventy-one primary research articles were reported across the 20 reviews. After removal of duplicates (*n* = 148), articles conducted in the wrong setting/population type/reporting the wrong outcomes (*n* = 471) and articles of low-quality (*n* = 72), moderate-quality (*n* = 51) or an unclear risk of bias (*n* = 6) were excluded, resulting in the inclusion of 23 primary research articles (studies) that implemented an in-store intervention designed to improve the healthiness of consumer purchasing or consumption (Figure 4).

#### 3.2.1 Study characteristics

Characteristics of the 23 primary studies included are presented in Appendix G. The studies were published between 1974 and 2022; about two thirds (*n* = 15 of 23, 65%) were published in or after 2000. Most (*n* = 14, 61%) were conducted in North America (69–82), six in the Pacific region (83–88), and three in Europe (89–91).

The number of stores included in primary studies ranged from one to 372. Most studies (*n* = 21 of 23, 91%) were conducted in physical (‘brick and mortar’) supermarket or grocery stores, and two studies used online supermarkets as the setting for their intervention. Regular shoppers (that is, no specific subgroup) were the target population for most studies (*n* = 16, 70%), five studies targeted low-income or food insecure individuals or communities, and two studies targeted minority groups including individuals living in regional or rural areas.

**Figure 5:**
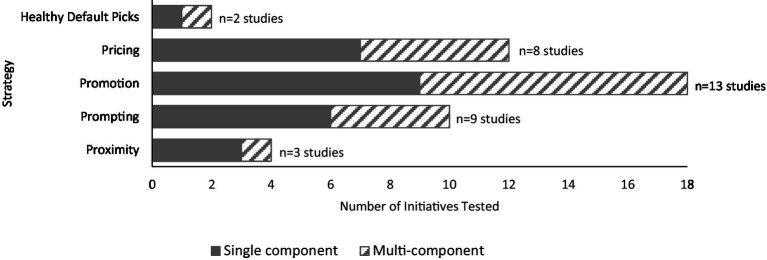
Distribution of strategies tested across the 23 primary studies. Studies may have included multiple intervention arms, therefore the number of initiatives presented in the graph may exceed the total number of primary studies included in the review.

There were 46 initiatives (categorised within five broad strategies) tested across the 23 studies (Figure 5). About half of the studies (*n* = 12, 52%) tested a single strategy, and the remaining studies (*n* = 11, 48%) tested multiple single strategies, or a combination of strategies (Appendix G). In-store promotion was the most frequently assessed intervention strategy (*n* = 13 of 23, 56%). Common promotion strategies included providing education to customers about the health benefits of selected products, offering samples of products and giving food demonstrations. Use of prompting was assessed in nine studies (39%), most commonly through in-store signage such as shelf labels and banners to identify healthier products. Pricing strategies were assessed in eight studies (35%), which included at the point of sale, via redeemable coupons or price reductions on target products, or after purchase via rebates. Use of proximity was assessed in three studies, and healthy default picks in two studies. No studies assessed place strategies, and, by design (i.e., per study eligibility criteria), no studies used profile, or portioning strategies. The duration of the interventions ranged between 2 h and 2 years. Just over a third of the studies (*n* = 9 of 23, 39%) included a follow-up period to ascertain the extent to which intervention effects were maintained after the intervention ended.

Customer purchasing behaviour was measured using either sales data, customer receipts, customer surveys, researcher observation, or a combination of these. Sales data were presented as total sales, sales/market share of target products, or expressed as healthiness of food purchases, such as energy density of foods purchased. In addition to consumer purchasing behaviour, four studies reported consumption of target products (via consumption questionnaires), and one study used a survey to collect information on skills and behaviours, such as food preparation practices and reading food labels.

#### 3.2.2 Study findings

Results from the primary studies are presented in Appendix H. All studies aimed to improve the healthiness of consumers’ purchases, and characterised products as healthy or unhealthy/less healthy. There is no consensus on the definitions of the terms healthy foods and unhealthy foods (92). In this review, the categorisation of foods and beverages into ‘healthy’ and ‘unhealthy/less healthy’ was taken from the description in the primary studies. In most studies (*n* = 21 of 23, 91%), the goal was to increase sales of healthy products, most commonly fruit and vegetables, or products with a higher nutritional ranking. Some of these studies (*n* = 6 of 21, 29%) also examined the effect on the sales of unhealthy/less healthy products (70, 71, 74, 84, 85, 87). Only two studies (9%) stated an intent to reduce sales of unhealthy/less healthy products (86, 90).

**Figure 6:**
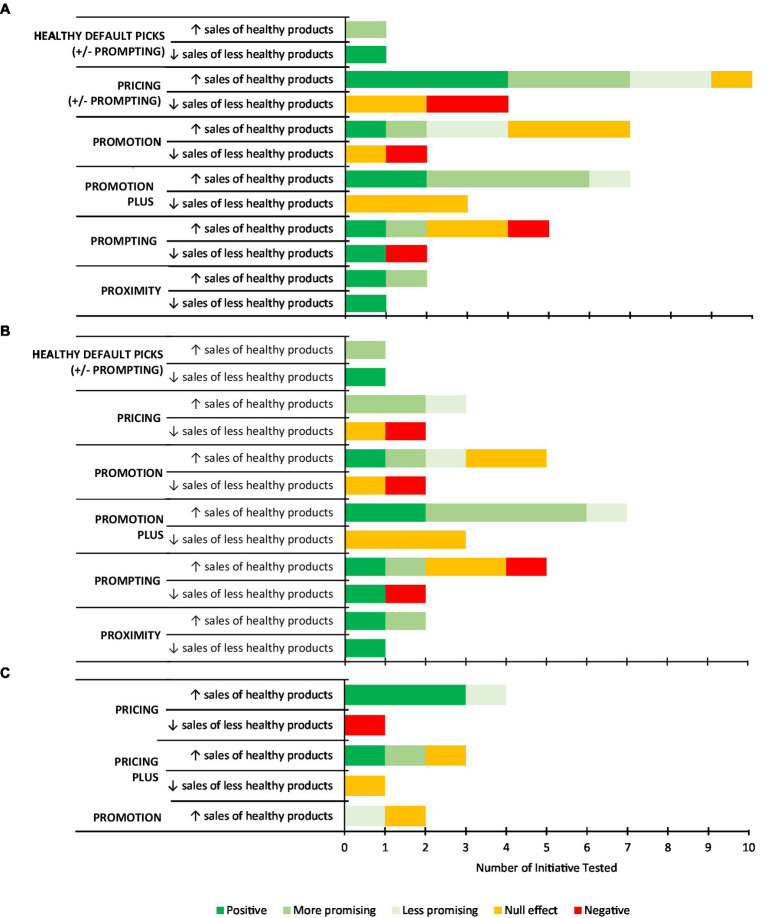
Summary of the effectiveness of primary studies included in the review at increasing the sales of healthy food products or decreasing the sales of less healthy food products, by strategy type among **(A)** the whole sample; **(B)** the general population; and **(C)** low SES or regional sub-groups. *Note:* Studies may have included multiple intervention arms with different initiatives or strategies/combinations of strategies, therefore the number of initiatives presented may exceed the total number of primary studies included in the review.

The effectiveness of intervention strategies on changing consumer purchasing of healthy and unhealthy products is summarised in Figure 6A. Studies considered as having ‘mixed’ findings were due to differences in the effectiveness of the intervention reported against multiple outcomes. For example, a significant effect may have been reported for purchase of fruit, but not vegetables. To aid interpretation, these mixed studies were separated into ‘more’ or ‘less’ promising, with the former representing cases where half or more of the categories assessed showed promise, or effects were not maintained over a longer period and vice versa for the latter (i.e., less promising). Given the purpose of the current synthesis was to inform strategies for targeting food purchasing among the general population, those studies that focussed on specific groups (low SES or regional) were separated from the synthesis of results and discussed independently.

#### 3.2.3 Characteristics of intervention strategies for decreasing purchasing of less healthy foods among the general population

Only three out of 10 initiatives (30%) were effective in decreasing sales of less healthy foods among the general population (Figure 6B). Two of these effective initiatives achieved their intended aim of decreasing sales of less healthy foods (86, 90), and the other decreased sales of less healthy foods as a consequence of the intervention aimed at promoting sales of healthier products. That is, 100% of studies (*n* = 2) that purposely aimed to reduce sales of unhealthy/less healthy products were effective.

Huang et al. (86) used *healthy default picks* to reduce sales of commonly purchased foods higher in saturated fat, particularly higher-fat dairy products, in an online supermarket setting. Customers were recommended different like-for-like product ‘swaps’, which were lower in saturated fat than the product they selected and were given the option to either retain the chosen product, or swap to the alternative. The amount of saturated fat (per cent of food) purchased by consumers in the intervention group decreased and lower-fat dairy products were the most common items ‘swapped’.

*Proximity* when applied at the checkout, that is, having “healthy checkouts” whereby unhealthy items such as sweets and chocolate were replaced with healthier options such as dried fruit, nuts, juices, and water was effective in reducing sales of less healthy foods (90). More explicit strategies such as promotion, pricing and prompting seemed to either be ineffective (*n* = 5 initiatives, 50%) or had negative effects (increase in sales of these products; *n* = 2 initiatives, 20%). There was, however, one exception based on a simple labelling system (71) that decreased sales of less healthy options, but was not effective at increasing sale of nutritious food, following implementation of a nutrition rating system on store shelves rating products with no-, one-, two-, or three-stars (Guiding Stars). In contrast, the labelling system that had a negative effect (increased sales of less healthy products) on sales was also the most complicated (70).

#### 3.2.4 Characteristics of intervention strategies for increasing healthy foods among the general population

Four out of 22 initiatives (18%) were effective in increasing sales of healthy foods among the general population, 13 (59%) were promising (*n* = 10 more promising, *n* = 3 less promising), four (18%) were ineffective and one (5%) reported negative effects (decreased sales of healthy products; Figure 6B). Promotion was the most common strategy assessed, followed by prompting, then pricing.

Supermarket nutrition education tours were the most effective *promotional* initiative in changing sales of healthy products (82). Following a 2-h dietitian-led supermarket tour where participants received advice about how to make sound nutrition choices (aimed at increasing intake of fibre and decreasing intakes of fat and salt,) participants reported that they purchased more healthier food options. However, overall, promotion alone appeared to have no or less promising effects. Three of the five (60%) *promotional* initiatives to improve consumer purchasing were ineffective (84, 87) or less promising (73). All studies included an educational component to support purchase of healthier products.

The combination of promotion with other strategies (*‘promotion plus’*) appeared to be the most favourable of the strategies considered, with five out of six studies (83%) using this approach either showing promise (78, 81, 84) or being effective (75, 87) at changing purchase of healthy items. Of these, three used a combination of *promotion and price—*with a price discount of between 10 and 50% (78, 84, 87), and two used *promotion and prompting* (75, 81). The promotional component of the interventions was similar, offering education to consumers about the nutritional content of foods via supermarket tours or provision of educational materials in the form of brochures and newsletters.

*Prompting* initiatives included the use of shelf-labels to support consumers to identify ‘better’ food choices across a range of products. Two of the five studies that used *prompting* as their intervention strategy were considered effective (77) or promising (76). One study reported an increase in purchase of healthier products across all eight categories of products tested, following the implementation of a nutrition scoring shelf-label system (NuVal) at the point-of-sale (77). Mixed findings were reported in a study that included a range of different products, whereby sales of some healthy products increased, or sales of less healthy products decreased, but others did not change (76). Prompting also resulted in two negative outcomes in the same study (70), namely a decrease in the sales of an item (popcorn) overall, but coupled with an increase in sales of the less healthy version of this same item. In other studies, there was no effect on the sale of fresh fruit and vegetables (69) or nutritious foods across a range of categories (71). Overall, prompting did not appear to be an effective strategy in the majority of studies conducted to date.

Curhan and colleagues (72) reported the effectiveness of two different *proximity* initiatives on increasing the sales of selected fruits and vegetables. ‘Bonus’ display space, that is, space allocation of at least 200% of the space usually allocated to products, increased the sales for all categories of fruits and vegetables (i.e., was effective). However, ‘location quality’, that is, high-traffic positions, increased the sales of some categories of fruits and vegetables (hard fruit and cooking vegetables), but not others (soft fruit or salad vegetables; i.e., was promising).

Another study in an online supermarket used *promotion and healthy default picks* centred around promoting images of healthier ‘like-for-like’ products on selected webpages (in-aisle banners and recipe bundles) (89). There was an increase in purchase of some healthier products, but not others.

*Pricing* had largely mixed effects across the three studies (72, 84, 87). Pricing initiatives reported improvements in selected discounted foods, but not others; however the food products and their effectiveness was inconsistent across studies.

#### 3.2.5 Characteristics of intervention strategies among low SES or regional subgroups

The effectiveness of intervention strategies on changing consumer purchasing of healthy and unhealthy products for population sub-groups are summarised in Figure 6C. Among low SES or regional sub-groups, *pricing* strategies were most assessed (4 out of 7 studies, 57%). Three studies reported effective *pricing* strategies and used discounts between 20 and 50% on selected food and drinks. Brimblecombe and colleagues (85) reported sales of healthy, and less healthy food and drinks, following a 24-week intervention which offered customers a 20% price reduction. The other two studies offered a 50% price reduction on fruit and vegetables at the point of sale via coupons (79) or after purchase, via rebates (91). Discounting had a negative effect on reducing less healthy foods, increasing purchases of sugar sweetened beverages (85). One study used three groups to compare pricing, promotion and the combination of both strategies in a group of low SES shoppers in the Netherlands (91). This study reported positive effects for price and price combined with promotion, but not promotion alone, which reported some, albeit less promising, changes in purchasing. Prompting was only considered in combination with promotion in this group of consumers from rural communities, with minimal effects on purchasing behaviour (80).

## 4 Discussion

Supermarkets have unprecedented and disproportionate power in the food system, influencing population diets through the products they have for sale, their price, store layouts and other marketing activities (93). In view of this, the World Health Organization advises governments worldwide to “develop policy measures that engage food retailers and caterers to improve the availability, affordability, and acceptability of healthier food products” (94). This review examined the effectiveness of strategies used in supermarket interventions to understand which strategies have shown promise in improving the healthfulness of consumer purchasing. Overall, the body of evidence reviewed shows that implementation of health promoting supermarket interventions are more likely to be successful if they include a substantial pricing initiative (particularly for some population sub-groups), or the inclusion of promotion in combination with another strategy.

Retailers need to consider their ‘bottom-line’ during implementation of any new initiative (95). Therefore, focusing on strategies to increase consumer purchasing would be more likely to be accepted and implemented by retailers. There were more interventions aimed at increasing sales (of healthy products) compared to decreasing sales (of less healthy products) in this review. There was also a higher success rate of interventions that aimed to decrease sales of less healthy products (100% were effective) than those that aimed to increase sales of healthy products (18% were effective). However, only two studies intentionally aimed to reduce sales of less healthy products, so there was not enough high-quality evidence to guide strategies to decrease purchase of less healthy food. The relative success or failure of initiatives may also be related to the type of product(s) selected as targets for intervention. In fact, retailers have previously identified lack of perceived consumer demand for healthy food, and a fear of profit loss as challenges (47). Findings from this review do not indicate a particular healthier food category was more successful than others. Some studies reported increased fruit but not vegetables, others increase in certain types of vegetable but not others, some increased low-fat dairy and others increased healthier tinned goods. Therefore, thought needs to be given not only to the strategies but also to the foods and beverages targeted.

Promotion was a popular strategy amongst papers reviewed, perhaps because of its relative influence in shaping consumer decisions in retail stores (96). Findings from this review highlight that, when used alone, the evidence for promotional initiatives is mixed. In contrast, when promotional initiatives are used in combination with another strategy, they produced favourable effects. Most promotional initiatives used in these studies focused on educating consumers about their food choices via provision of materials in the form of brochures and newsletters, or in-store demonstrations including taste-tests and supermarket tours. Nutrition education and knowledge has been shown to influence consumers ability to identify healthy foods (97), but this does not necessarily alter intentions or behaviour (98). In fact, findings from an umbrella review of food choice and nutrition support the findings of this review, suggesting that combining strategies appears to be the most effective way to achieve healthier food choices (98). It was not possible to determine which combination with promotion was most effective, mainly due to the small number of studies. Promotion and price were used together in three studies and promotion and prompting in two studies, and in both combinations one study reported positive outcomes.

Of the strategies evaluated here, pricing, whether combined with another strategy or tested on its own, appeared to be the most promising strategy at increasing sales of healthy products. The relative success of a pricing initiative does not appear to be strongly influenced by the magnitude of the discount. Discounts applied in successful pricing initiatives ranged between 20 and 50%, and 10 to 50% for unsuccessful initiatives. This is in contrast to economic research that suggests that consumers do not change their intentions to buy unless the promotional discount is above a threshold level (99). Pricing initiatives were more successful among studies that included shoppers in rural or remote areas, or those from low-income households, which is consistent with our understanding that greater affordability/access leads to increased consumption of discounted products, particularly among food insecure groups (100). More research is needed to understand whether all segments of the population benefit from pricing initiatives, the magnitude of the change in price needed to influence consumer purchasing, if there is a saturation point above which, the effect of discounts is minimal, and if such substantial discounts reported in the literature are sustainable for retailers in the long-term.

There was a modest proportion of negative findings (i.e., results going in the opposite direction to that intended) reported among studies reviewed that aimed to increase sales of healthy products (3 out of 6, 50%). Compensatory purchasing can be a problematic side effect of pricing initiatives (101). For example, when discounting healthy food, savings may be used to buy more less healthy products, as observed in two studies in the review (84, 85). Similarly, promoting healthy items next to unhealthy items may also have unintended effects (e.g., water and sugar-sweetened beverages). The implementation of a ‘swap’ message for popcorn was also associated with an unintended outcome in one of the studies included; while the intervention resulted in less popcorn being sold overall, it was also associated with a shift towards consumption of less healthy popcorn varieties, at the expense of the healthier alternatives (70). These findings highlight the importance considering and evaluating the unintended consequences for retailers, consumers, and the broader community, when implementing new initiatives in a supermarket setting. This includes measuring sales of all products purchased, not just of targeted products, and measuring outcomes beyond sales. Blake and colleagues (52) use a scoping review to summarise the types of business outcomes used in healthy food and beverage retail strategies, including outcomes that may affect retailers’ likelihood of implementing and sustaining a healthy food retail strategy—namely, commercial viability, customer and retailer perspectives, and community outcomes. In general, the selection of business outcomes and measurement tools could be chosen in consultation with the retailer, considering feasibility, and the marginal cost and value of adjusting nutrition data collection methods (e.g., including questions on customer level of satisfaction in a survey focusing on changes in consumption). Consideration of the types of business outcomes that are most relevant to different strategies and settings may allow for more tailored data collection in future studies.

Interventions in supermarkets are often implemented over a short period and/or in a single store, with little attention placed on the long-term sustainability or scalability of the interventions. Less than half of the studies in this review included a follow-up period (range 4 weeks to 104 weeks, average ~ 6 months) to ascertain the extent to which intervention effects were maintained after the intervention ended. Of those that included a follow-up period, about half found that some effects were maintained after removal of the support. For population dietary change that is sustainable in the longer-term, initiatives in supermarkets need to be both feasible for retailers, and acceptable to consumers. In their overview of reviews investigating the factors that influence the implementation, sustainability and scalability of healthy food retail interventions, Gupta and colleagues (47) emphasise the importance of considering how contextual barriers, such as food store structure, low consumer demand and reduced sales or profitability, may be linked to retailers’ perceptions, to increase the likelihood of sustained implementation and for potential scale up.

### 4.1 Strengths and limitations

Strengths of this study include a comprehensive search strategy that was developed (in collaboration with an experienced librarian) and adapted for seven databases to best capture all available evidence. The study also observed PRIOR/PRISMA guidelines with the protocol pre-registered on OSF and deviations disclosed. Screening processes and the risk of bias appraisal were conducted by two reviewers independently. Well-defined study selection criteria and independent coding of the findings make this review process rigorous and robust. Another strength of this study is the inclusion of studies with greater external validity – only those conducted in real-world physical or online supermarket settings and excluded simulation or laboratory studies. Only two studies included were conducted in online supermarkets, so little is known about the effectiveness of initiatives in this emerging food retail setting. The novel approach to identify strong primary studies from strong reviews also meant that a large amount of literature could be assessed without losing detail about what strategies show promise.

Some limitations to this study must also be acknowledged. Firstly, findings from the overview of reviews were restricted to the analyses reported in the included reviews. There was some duplication of primary studies across the reviews, which may have led to some heterogeneity in the findings within the individual reviews, as well as in the overview of reviews (Part I). Secondly, primary studies evaluated in the review (Part II) were identified from the overview of reviews. As such, there may be gaps in the evidence base for some intervention strategies due to study selection, rather than an absence of primary studies. This also means current, primary studies were overlooked because they have not yet been included in reviews. The decision to include only high-quality primary studies meant a higher degree of confidence in study findings, but it could also be considered a limitation. Across the reviews, a range of tools were used by the original review authors to appraise the quality of primary studies, resulting in conflicting quality ratings of studies, potentially due to differences in aspects covered in the tools. These reviews were rated high-quality by the current process, so it was assumed their evaluations of other studies would be also be acceptable. Furthermore, some studies that scored low in the methodological quality may have other strengths not accounted for by the respective scoring systems. Thirdly, publication bias cannot be excluded; ineffective interventions are less likely to be published, and only articles published in English language were included, which may have led to exclusion of relevant reviews and primary studies. Finally, most studies included in this review only measured sales of products, with few studies measuring both sales and consumption. Although sales can be considered a proxy for consumption, it cannot be concluded that increasing sales of selected products led to greater consumption.

## 5 Conclusion

Food retailers are a key influence of population diets. Stakeholder engagement and use of the right incentives are essential to the success of the interventions and their sustainability longer-term. Therefore, it is critical to optimise the potential and power of supermarket retailers by working with them to make sustainable and scalable changes that help consumers to make purchases that preference healthier foods, without significantly impacting their bottom line. The current study identifies a range of initiatives to improve consumer purchasing behaviour. Owing to the heterogeneous nature of the study exposures, interventions, and outcomes, it is difficult to draw definitive conclusions from the available, published evidence, and few studies included a follow-up period, so even less is known about the longer-term sustainability of these initiatives. Promotional strategies paired with another strategy appear promising for increasing sales of healthy foods. Pricing strategies also have promise, however, the amount price needs to change to influence consumer purchasing and to produce meaningful changes in measures related to public health, and their effectiveness outside of particular sub-groups, should be explored further.

## Data availability statement

The original contributions presented in the study are included in the article/Supplementary material, further inquiries can be directed to the corresponding author/s.

## Author contributions

PB: Conceptualization, Data curation, Formal analysis, Methodology, Project administration, Writing – original draft, Writing – review & editing. CH: Conceptualization, Data curation, Formal analysis, Methodology, Writing – review & editing. EB: Conceptualization, Formal analysis, Methodology, Writing – review & editing. GH: Conceptualization, Formal analysis, Funding acquisition, Methodology, Writing – review & editing.

## Footnotes

^1^ https://osf.io/jyg73/
