# Supplementary material for: Strategies associated with improved healthiness of consumer purchasing in supermarket interventions: a systematic overview of reviews and evaluation of primary articles
Source: Front Public Health. 2024 Jun 25;12:1334324. doi: 10.3389/fpubh.2024.1334324 (PMC11232481; doi:10.3389/fpubh.2024.1334324)
Supplement: Supplementary file 1 [file Table_1.DOCX]

**Brooker, PG, Howlett, CA, Brindal, E & Hendrie, GA. Strategies associated with improved healthiness of consumer purchasing in supermarket interventions: a systematic overview of reviews and an evaluation of primary articles**

**Corresponding author:**

Paige G Brooker

Address: Health and Biosecurity, Commonwealth Scientific and Industrial Research Organisation (CSIRO), PO BOX 10041 Adelaide BC 5000, South Australia, Australia

E: paige.brooker@csiro.au

**Appendix A: Pre-registered study protocol**

**Objective/aim**

To synthesise and evaluate the evidence on initiatives in the food retail sector that aim to improve the healthiness of food and beverage purchases and/or consumption.

**Methods**

The Preferred Reporting Items for Overviews of Reviews (PRIOR) guidelines will be adhered to during the implementation and reporting of this study.

**Inclusion Criteria**

- *Design:* Full-text review articles (scoping, systematic, narrative, umbrella).
- *Population:* Regular shoppers who can make purchases independently (i.e., adults and adolescents).
- *Setting:* Real-world (i.e., physical, or online, hybrid) supermarkets or grocery stores where a transaction occurs in exchange for food and beverage items to be consumed within the home environment. Reviews that include primary studies conducted in other retail settings such as vending machines, cafeterias, corner stores, famer’s markets etc) will be included if they also include supermarkets and/or grocery stores.
- *Intervention/exposure:* Real-world intervention strategies that aims to improve the healthiness of the non-alcoholic in-store food and beverage environment. May include merchandising strategies focusing on, for example, product (e.g., availability) price (e.g., promotions), promotion (e.g., advertising), placement, or a combination of these.
- *Comparator:* A comparator will be included if reported in the included reviews.
- *Outcome:* Purchase-related outcomes – either objective (e.g., sales data, customer receipts), or subjective (e.g., observation); consumption-related outcomes (e.g., dietary recalls); health-related outcomes (e.g., weight, BMI); or food environment outcomes (e.g., store audit, customer surveys, food traffic).

**Exclusion Criteria**

- *Design:* Primary research articles
- *Population:* Children or persons who do not purchase food products independently.
- *Setting:* Any food retail environment where providing food is not the primary purpose (e.g., service stations, schools), environments where food is prepared and sold only in a read-to-eat format (e.g., restaurants, fast-food services, vending machines etc), other small retailers (e.g., corner/convenience stores, farmer’s markets etc), or the opening of new stores. Any intervention that does not replicate a real-world shopping experience (i.e., where no money is exchanged), such as simulation/laboratory studies.
- *Intervention/exposure:* Strategies outside of the retailers’ control, such as changes to front/back-of-pack labelling, taxation, government subsides, access to stores, built environment (proximity of stores), or reformulation.
- *Comparator:* Not applicable.
- *Outcome:* Any outcome that does not focus on adopting healthier food purchases or consumption, or does not impact health (e.g., hypothetical choice/intention to purchase, shelf-space). Additionally, any outcomes related to the purchases and/or consumption of alcoholic beverages are beyond the scope of this study.

**Search strategy**

The following electronic databases will be searched: Pubmed, Medline, Scopus, Web of Science and Google Scholar. A search strategy will be developed using MeSH headings and keywords such as ‘effectiveness’, ‘interventions’, ‘food retail’, ‘food purchase’, and ‘consumption’. Databases will be searched from inception and will be restricted to reviews published in English; no other restrictions will be applied. Reference lists of relevant reviews will be manually searched to capture any additional citations missed by electronic database searches. Additional review articles obtained will be subject to the same selection criteria as other articles.

**Data extraction**

All citations will be imported into Endnote and duplicates will be removed. All de-duplicated citations will then be imported into Covidence software. Title and abstract screening will be conducted to identify reviews meeting the inclusion and exclusion criteria. Any reviews not meeting the inclusion/exclusion criteria will be excluded. Two independent reviewers will complete this process and any discrepancies will be resolved via discussion. Where consensus is unable to be reached, a third reviewer will be consulted to make the final decision. Data from eligible reviews will be extracted using an Excel spreadsheet. The following information will be extracted: publication details (author, title, year of publication, type of review and number of studies included in the review, aim), intervention components (type of food retailer, setting, time period, intervention design, target foods), context (including intervention designers, stakeholders involved), measures of effectiveness; and outcomes. Where relevant data are missing or not reported, we will attempt to contact the review authors to try to obtain such information.

Following this, high quality original research articles (as assessed by the original review authors) will be sourced from each high quality reviews included in our umbrella review. Data extraction will be repeated as per the above.

**Quality appraisal/risk of bias assessment**

All reviews will be assessed for methodological quality by two independent reviewers using either the Risk of Bias in Systematic Reviews (ROBIS) tool or A Measurement Tool to Assess the Methodological Quality of Systematic Reviews (AMSTAR). Any discrepancies that arise will be resolved through discussion, or where consensus cannot be established, a third reviewer will be consulted to make the final decision.

**Data synthesis**

Due to the expected heterogeneity among reviews, a narrative synthesis of findings for will be performed.

**Appendix B: Search strategy**

Table S1. Literature Search Strategy

| **Database searched**  **[date]** | **Search Terms** | **Filters / Limiters applied** |
| --- | --- | --- |
| **PubMed**  **[31.03.2023]** | ("store tour*"[Title/Abstract] OR intervention*[Title/Abstract] OR "choice architecture"[Title/Abstract] OR promotion[Title/Abstract] OR incentive*[Title/Abstract] OR disincentive*[Title/Abstract] OR priming[Title/Abstract] OR discount*[Title/Abstract] OR price[Title/Abstract] OR pricing[Title/Abstract] OR voucher[Title/Abstract] OR bonus[Title/Abstract] OR reward*[Title/Abstract] OR coupon*[Title/Abstract] OR token*[Title/Abstract] OR rebate*[Title/Abstract] OR refund*[Title/Abstract] OR reimburse*[Title/Abstract] OR display[Title/Abstract] OR remov*[Title/Abstract] OR layout[Title/Abstract] OR strateg*[Title/Abstract] OR advert*[Title/Abstract] OR market[Title/Abstract] OR marketing[Title/Abstract] OR initiative*[Title/Abstract] OR program*[Title/Abstract] OR "loyalty card*"[Title/Abstract] OR "point of purchase"[Title/Abstract] OR "point of sale"[Title/Abstract] OR "point of choice"[Title/Abstract] OR "product placement"[Title/Abstract] OR "food placement"[Title/Abstract] OR "shelf placement"[Title/Abstract] OR proximity[Title/Abstract] OR positioning[Title/Abstract] OR "retail environment*"[Title/Abstract] OR "food environment"[Title/Abstract] OR "product swap"[Title/Abstract:~0] OR "product signage"[Title/Abstract:~0] OR "product availability"[Title/Abstract] OR "store layout"[Title/Abstract] OR "checkout area"[Title/Abstract] OR "store entrance"[Title/Abstract] OR "floor display"[Title/Abstract] OR "product recommendation"[Title/Abstract] OR advertising[Title/Abstract] OR advertisement[Title/Abstract]) OR ("Marketing"[Mesh] OR "Overweight/prevention and control"[Mesh] OR "Obesity/prevention and control"[Mesh] OR "Advertising"[Mesh] OR "Direct to Consumer Advertising"[Mesh] OR "Health Promotion"[Mesh] OR "Health Education"[Mesh] OR "Preventive Medicine"[Mesh:NoExp] OR "Health Plan Implementation"[Mesh]) AND (Food*[Title/Abstract] OR groceries[Title/Abstract] OR snack*[Title/Abstract] OR fruit*[Title/Abstract] OR vegetable*[Title/Abstract] OR nuts[Title/Abstract] OR beverage*[Title/Abstract] OR legumes[Title/Abstract] OR drink[Title/Abstract] OR drinks[Title/Abstract] OR nutrition[Title/Abstract] OR "Food"[Mesh] OR "Food Industry"[Mesh] OR "Beverages"[Mesh] OR "Diet"[Mesh]) AND ("dietary outcome"[Title/Abstract] OR "dietary-related outcome"[Title/Abstract:~0] OR "health benefit"[Title/Abstract] OR "dietary intake"[Title/Abstract] OR "diet quality"[Title/Abstract] OR "eating behaviour*"[Title/Abstract] OR "eating behavior*"[Title/Abstract] OR "healthy eating"[Title/Abstract] OR "healthy diet"[Title/Abstract] OR Healthy[Title/Abstract] OR healthier[Title/Abstract] OR healthiness[Title/Abstract] OR unhealthy[Title/Abstract] OR "low fat"[Title/Abstract] OR "low sodium"[Title/Abstract] OR "low sugar"[Title/Abstract] OR low-fat[Title/Abstract] OR low-sodium[Title/Abstract] OR low-sugar[Title/Abstract] OR purchas*[Title/Abstract] OR consumption[Title/Abstract] OR "sales data"[Title/Abstract] OR "sales outcome*"[Title/Abstract] OR "customer satisfaction"[Title/Abstract] OR "sales effect"[Title/Abstract:~0] OR "sales effects"[Title/Abstract:~0] OR "business outcome*"[Title/Abstract] OR revenue[Title/Abstract] OR profitability[Title/Abstract] OR profit[Title/Abstract] OR margin[Title/Abstract] OR turnover[Title/Abstract] OR ROI[Title/Abstract] OR cost-benefit[Title/Abstract] OR "cost benefit"[Title/Abstract] OR "return on investment"[Title/Abstract] OR "item sales"[Title/Abstract] OR "total sales"[Title/Abstract] OR "commercial viability"[Title/Abstract] OR "opportunity cost"[Title/Abstract] OR opportunity-cost[Title/Abstract] OR value-proposition[Title/Abstract] OR "value proposition"[Title/Abstract] OR "foot traffic"[Title/Abstract] OR patronage[Title/Abstract] OR "customer loyalty"[Title/Abstract] OR "store loyalty"[Title/Abstract] OR "return customer"[Title/Abstract:~0] OR "return customers"[Title/Abstract:~0] OR "customer demand"[Title/Abstract] OR BMI[Title/Abstract] OR bodyweight[Title/Abstract] OR "body weight"[Title/Abstract] OR overweight[Title/Abstract] OR "muscle mass"[Title/Abstract] OR "lean mass"[Title/Abstract] OR obesity[Title/Abstract] OR "weight gain"[Title/Abstract] OR "weight loss"[Title/Abstract] OR adiposity[Title/Abstract] OR "Consumer Behavior"[Mesh] OR "Choice Behavior"[Mesh:NoExp] OR "Motivation"[Mesh] OR "Food Preferences"[Mesh] OR "Program Evaluation"[Mesh] OR "Attitude to Health"[Mesh] OR "Drinking Behavior"[Mesh] OR "Costs and Cost Analysis"[Mesh] OR "Diet, Healthy"[Mesh] OR "Economics, Behavioral"[Mesh] OR "Body Mass Index"[Mesh] OR "Obesity"[Mesh] OR "Overweight"[Mesh] OR "Weight Loss"[Mesh] OR "Weight Gain"[Mesh] OR "Adiposity"[Mesh]) AND (Supermarket*[Title/Abstract] OR "grocery store*"[Title/Abstract] OR "food store*"[Title/Abstract] OR grocer*[Title/Abstract] OR hypermarket*[Title/Abstract] OR bodega*[Title/Abstract] OR "convenience store*"[Title/Abstract] OR "food market"[Title/Abstract] OR "food retail*"[Title/Abstract] OR “retail food”[Title/Abstract] OR Supermarkets[Mesh]) AND ("systematic review"[Title/Abstract] OR "scoping review"[Title/Abstract] OR metanalysis[Title/Abstract] OR metanalyses[Title/Abstract] OR meta-analysis[Title/Abstract] OR meta-analyses[Title/Abstract] OR "narrative review" "literature review"[Title/Abstract] OR "rapid review"[Title/Abstract] OR "critically appraised topic"[Title/Abstract] OR overview[Title/Abstract] OR "collaborative review"[Title/Abstract] OR "umbrella review"[Title/Abstract] OR handsearch[Title/Abstract] OR "hand search"[Title/Abstract] OR "data synthesis"[Title/Abstract] OR "data extraction"[Title/Abstract] OR "Meta-Analysis"[Publication Type] OR "Review"[Publication Type] OR "Systematic Review"[Publication Type] OR "Meta-Analysis as Topic"[Mesh] OR "Review Literature as Topic"[Mesh] OR "Systematic Reviews as Topic"[Mesh] OR "Systematic Reviews as Topic"[Mesh]) | none |
| **Web of Knowledge Core Collection**  **[31.03.2023]** | ((TI="store tour*" OR AB="store tour*") OR (TI=intervention* OR AB=intervention*) OR (TI="choice architecture" OR AB="choice architecture") OR (TI=promotion OR AB=promotion) OR (TI=incentive* OR AB=incentive*) OR (TI=disincentive* OR AB=disincentive*) OR (TI=priming OR AB=priming) OR (TI=discount* OR AB=discount*) OR (TI=price OR AB=price) OR (TI=pricing OR AB=pricing) OR (TI=voucher OR AB=voucher) OR (TI=bonus OR AB=bonus) OR (TI=reward* OR AB=reward*) OR (TI=coupon* OR AB=coupon*) OR (TI=token* OR AB=token*) OR (TI=rebate* OR AB=rebate*) OR (TI=refund* OR AB=refund*) OR (TI=reimburse* OR AB=reimburse*) OR (TI=display OR AB=display) OR (TI=remov* OR AB=remov*) OR (TI=layout OR AB=layout) OR (TI=strateg* OR AB=strateg*) OR (TI=advert* OR AB=advert*) OR (TI=market OR AB=market) OR (TI=Marketing OR AB=Marketing) OR (TI=initiative* OR AB=initiative*) OR (TI=program* OR AB=program*) OR (TI="loyalty card*" OR AB="loyalty card*") OR (TI="point of purchase" OR AB="point of purchase") OR (TI="point of sale" OR AB="point of sale") OR (TI="point of choice" OR AB="point of choice") OR (TI="product placement" OR AB="product placement") OR (TI="food placement" OR AB="food placement") OR (TI="shelf placement" OR AB="shelf placement") OR (TI=proximity OR AB=proximity) OR (TI=positioning OR AB=positioning) OR (TI="retail environment*" OR AB="retail environment*") OR (TI="food environment" OR AB="food environment") OR (TI="product swap*” OR AB=”product swap*) OR (TI="product signage” OR AB=”product signage”) OR (TI="product availability" OR AB="product availability") OR (TI="store layout" OR AB="store layout") OR (TI="checkout area" OR AB="checkout area") OR (TI="store entrance" OR AB="store entrance") OR (TI="floor display" OR AB="floor display") OR (TI="product recommendation" OR AB="product recommendation") OR (TI=Advertising OR AB=Advertising) OR (TI=advertisement OR AB=advertisement) OR (TI="Health Education" OR AB=”Health Education”) OR (AB="Preventive Medicine" OR TI=”Preventive Medicine”) OR (TI="Health Plan Implementation" OR AB=”Health Plan Implementation”)) AND ((TI=food* OR AB=food*) OR (TI=groceries OR AB=groceries) OR (TI=snack* OR AB=snack*) OR (TI=fruit* OR AB=fruit*) OR (TI=vegetable* OR AB=vegetable*) OR (TI=nuts OR AB=nuts) OR (TI=beverage* OR AB=beverage*) OR (TI=legumes OR AB=legumes) OR (TI=drink OR AB=drink) OR (TI=drinks OR AB=drinks) OR (TI=nutrition OR AB=nutrition) OR (TI=diet OR AB=diet)) AND ((TI="dietary outcome" OR AB="dietary outcome") OR ALL="dietary-related outcome[Title" OR (TI="health benefit" OR AB="health benefit") OR (TI="dietary intake" OR AB="dietary intake") OR (TI="diet quality" OR AB="diet quality") OR (TI="eating behaviour*" OR AB="eating behaviour*") OR (TI="eating behavior*" OR AB="eating behavior*") OR (TI="healthy eating" OR AB="healthy eating") OR (TI="healthy diet" OR AB="healthy diet") OR (TI=Healthy OR AB=Healthy) OR (TI=healthier OR AB=healthier) OR (TI=healthiness OR AB=healthiness) OR (TI=unhealthy OR AB=unhealthy) OR (TI=low-fat OR AB=low-fat) OR (TI=low-sodium OR AB=low-sodium) OR (TI=low-sugar OR AB=low-sugar) OR (TI=low-fat OR AB=low-fat) OR (TI=low-sodium OR AB=low-sodium) OR (TI=low-sugar OR AB=low-sugar) OR (TI=purchas* OR AB=purchas*) OR (TI=consumption OR AB=consumption) OR (TI="sales data" OR AB="sales data") OR (TI="sales outcome*" OR AB="sales outcome*") OR (TI="customer satisfaction" OR AB="customer satisfaction") OR (TI="sales effect*” OR AB=”sales effect*) OR (TI="business outcome*" OR AB="business outcome*") OR (TI=revenue OR AB=revenue) OR (TI=profitability OR AB=profitability) OR (TI=profit OR AB=profit) OR (TI=margin OR AB=margin) OR (TI=turnover OR AB=turnover) OR (TI=ROI OR AB=ROI) OR (TI=cost-benefit OR AB=cost-benefit) OR (TI=cost-benefit OR AB=cost-benefit) OR (TI="return on investment" OR AB="return on investment") OR (TI="item sales" OR AB="item sales") OR (TI="total sales" OR AB="total sales") OR (TI="commercial viability" OR AB="commercial viability") OR (TI=opportunity-cost OR AB=opportunity-cost) OR (TI=opportunity-cost OR AB=opportunity-cost) OR (TI=value-proposition OR AB=value-proposition) OR (TI=value-proposition OR AB=value-proposition) OR (TI="foot traffic" OR AB="foot traffic") OR (TI=patronage OR AB=patronage) OR (TI="customer loyalty" OR AB="customer loyalty") OR (TI="store loyalty" OR AB="store loyalty") OR (TI="return customer*” OR AB=”return customer*”) OR (TI="customer demand" OR AB="customer demand") OR (TI=BMI OR AB=BMI) OR (TI=bodyweight OR AB=bodyweight) OR (TI="body weight" OR AB="body weight") OR (TI=Overweight OR AB=Overweight) OR (TI="muscle mass" OR AB="muscle mass") OR (TI="lean mass" OR AB="lean mass") OR (TI=Obesity OR AB=Obesity) OR (TI="Weight Gain" OR AB="Weight Gain") OR (TI="Weight Loss" OR AB="Weight Loss") OR (TI=Adiposity OR AB=Adiposity) OR (TI="Consumer Behavi*" OR AB="Consumer Behavi*") OR (TI="Choice Behavior" OR AB="Choice Behavi") OR (TI=Motivation OR AB=Motivation) OR (TI="Food Preference*" OR AB="Food Preference*") OR (TI="Program Evaluation" OR AB=”Program Evaluation”) OR (TI="Attitude to Health" OR AB=”Attitude to Health”) OR (TI="Drinking Behavi*" OR AB="Drinking Behavi*") OR (TI=" Cost Analysis" OR AB=”Cost Analysis”) OR (TI="Body Mass Index" OR AB=”Body Mass Index”)) AND ((TI=supermarket* OR AB=supermarket*) OR (TI="grocery store*" OR AB="grocery store*") OR (TI="food store*" OR AB="food store*") OR (TI=grocer* OR AB=grocer*) OR (TI=hypermarket* OR AB=hypermarket*) OR (TI=bodega* OR AB=bodega*) OR (TI="convenience store*" OR AB="convenience store*") OR (TI="food market*" OR AB="food market*") OR (TI="food retail*" OR AB="food retail*") OR (TI="retail food" OR AB="retail food") OR ALL="retail store*" OR (TI="food vendor*" OR AB="food vendor*")) AND ((((TI="Systematic Review" OR AB="Systematic Review") OR (TI="scoping review" OR AB="scoping review") OR (TI=metanalysis OR AB=metanalysis) OR (TI=metanalyses OR AB=metanalyses) OR (TI="meta analysis" OR AB="meta analysis") OR (TI=meta-analyses OR AB=meta-analyses) OR (TI="narrative review" OR AB=”narrative review”) AND (TI="literature review" OR AB="literature review")) OR (TI="rapid review" OR AB="rapid review") OR (TI="critically appraised topic" OR AB="critically appraised topic") OR (TI=overview OR AB=overview) OR (TI="collaborative review" OR AB="collaborative review") OR (TI="umbrella review" OR AB="umbrella review") OR (TI=handsearch OR AB=handsearch) OR (TI="hand search" OR AB="hand search") OR (TI="data synthesis" OR AB="data synthesis") OR (TI="data extraction" OR AB="data extraction") OR (TI=”review literature” OR AB=”review literature”))) | none |
| **Scopus**  **[04.04.2023]** | ( TITLE-ABS ( "store tour*" ) OR TITLE-ABS ( intervention* ) OR TITLE-ABS ( "choice architecture" ) OR TITLE-ABS ( promotion ) OR TITLE-ABS ( incentive* ) OR TITLE-ABS ( disincentive* ) OR TITLE-ABS ( priming ) OR TITLE-ABS ( discount* ) OR TITLE-ABS ( price ) OR TITLE-ABS ( pricing ) OR TITLE-ABS ( voucher ) OR TITLE-ABS ( bonus ) OR TITLE-ABS ( reward* ) OR TITLE-ABS ( coupon* ) OR TITLE-ABS ( token* ) OR TITLE-ABS ( rebate* ) OR TITLE-ABS ( refund* ) OR TITLE-ABS ( reimburse* ) OR TITLE-ABS ( display ) OR TITLE-ABS ( remov* ) OR TITLE-ABS ( layout ) OR TITLE-ABS ( strateg* ) OR TITLE-ABS ( advert* ) OR TITLE-ABS ( market ) OR TITLE-ABS ( marketing ) OR TITLE-ABS ( initiative* ) OR TITLE-ABS ( program* ) OR TITLE-ABS ( "loyalty card*" ) OR TITLE-ABS ( "point of purchase" ) OR TITLE-ABS ( "point of sale" ) OR TITLE-ABS ( "point of choice" ) OR TITLE-ABS ( "product placement" ) OR TITLE-ABS ( "food placement" ) OR TITLE-ABS ( "shelf placement" ) OR TITLE-ABS ( proximity ) OR TITLE-ABS ( positioning ) OR TITLE-ABS ( "retail environment*" ) OR TITLE-ABS ( "food environment" ) OR INDEXTERMS ( "product swap[Title" ) OR INDEXTERMS ( "product signage[Title" ) OR TITLE-ABS ( "product availability" ) OR TITLE-ABS ( "store layout" ) OR TITLE-ABS ( "checkout area" ) OR TITLE-ABS ( "store entrance" ) OR TITLE-ABS ( "floor display" ) OR TITLE-ABS ( "product recommendation" ) OR TITLE-ABS ( advertising ) OR TITLE-ABS ( advertisement ) OR ( INDEXTERMS ( marketing ) OR INDEXTERMS ( "overweight/prevention and control" ) OR INDEXTERMS ( "obesity/prevention and control" ) OR INDEXTERMS ( advertising ) OR INDEXTERMS ( "Direct to Consumer Advertising" ) OR INDEXTERMS ( "Health Promotion" ) OR INDEXTERMS ( "Health Education" ) OR INDEXTERMS ( "Preventive Medicine" ) OR INDEXTERMS ( "Health Plan Implementation" ) ) ) AND ( TITLE-ABS ( food* ) OR TITLE-ABS ( groceries ) OR TITLE-ABS ( snack* ) OR TITLE-ABS ( fruit* ) OR TITLE-ABS ( vegetable* ) OR TITLE-ABS ( nuts ) OR TITLE-ABS ( beverage* ) OR TITLE-ABS ( legumes ) OR TITLE-ABS ( drink ) OR TITLE-ABS ( drinks ) OR TITLE-ABS ( nutrition ) OR INDEXTERMS ( food ) OR INDEXTERMS ( "Food Industry" ) OR INDEXTERMS ( beverages ) OR INDEXTERMS ( diet ) ) AND ( TITLE-ABS ( "dietary outcome" ) OR INDEXTERMS ( "dietary-related outcome[Title" ) OR TITLE-ABS ( "health benefit" ) OR TITLE-ABS ( "dietary intake" ) OR TITLE-ABS ( "diet quality" ) OR TITLE-ABS ( "eating behaviour*" ) OR TITLE-ABS ( "eating behavior*" ) OR TITLE-ABS ( "healthy eating" ) OR TITLE-ABS ( "healthy diet" ) OR TITLE-ABS ( healthy ) OR TITLE-ABS ( healthier ) OR TITLE-ABS ( healthiness ) OR TITLE-ABS ( unhealthy ) OR TITLE-ABS ( low-fat ) OR TITLE-ABS ( low-sodium ) OR TITLE-ABS ( low-sugar ) OR TITLE-ABS ( low-fat ) OR TITLE-ABS ( low-sodium ) OR TITLE-ABS ( low-sugar ) OR TITLE-ABS ( purchas* ) OR TITLE-ABS ( consumption ) OR TITLE-ABS ( "sales data" ) OR TITLE-ABS ( "sales outcome*" ) OR TITLE-ABS ( "customer satisfaction" ) OR INDEXTERMS ( "sales effect[Title" ) OR INDEXTERMS ( "sales effects[Title" ) OR TITLE-ABS ( "business outcome*" ) OR TITLE-ABS ( revenue ) OR TITLE-ABS ( profitability ) OR TITLE-ABS ( profit ) OR TITLE-ABS ( margin ) OR TITLE-ABS ( turnover ) OR TITLE-ABS ( roi ) OR TITLE-ABS ( cost-benefit ) OR TITLE-ABS ( cost-benefit ) OR TITLE-ABS ( "return on investment" ) OR TITLE-ABS ( "item sales" ) OR TITLE-ABS ( "total sales" ) OR TITLE-ABS ( "commercial viability" ) OR TITLE-ABS ( opportunity-cost ) OR TITLE-ABS ( opportunity-cost ) OR TITLE-ABS ( value-proposition ) OR TITLE-ABS ( value-proposition ) OR TITLE-ABS ( "foot traffic" ) OR TITLE-ABS ( patronage ) OR TITLE-ABS ( "customer loyalty" ) OR TITLE-ABS ( "store loyalty" ) OR INDEXTERMS ( "return customer[Title" ) OR INDEXTERMS ( "return customers[Title" ) OR TITLE-ABS ( "customer demand" ) OR TITLE-ABS ( bmi ) OR TITLE-ABS ( bodyweight ) OR TITLE-ABS ( "body weight" ) OR TITLE-ABS ( overweight ) OR TITLE-ABS ( "muscle mass" ) OR TITLE-ABS ( "lean mass" ) OR TITLE-ABS ( obesity ) OR TITLE-ABS ( "Weight Gain" ) OR TITLE-ABS ( "Weight Loss" ) OR TITLE-ABS ( adiposity ) OR INDEXTERMS ( "Consumer Behavior" ) OR INDEXTERMS ( "Choice Behavior" ) OR INDEXTERMS ( motivation ) OR INDEXTERMS ( "Food Preferences" ) OR INDEXTERMS ( "Program Evaluation" ) OR INDEXTERMS ( "Attitude to Health" ) OR INDEXTERMS ( "Drinking Behavior" ) OR INDEXTERMS ( "Costs and Cost Analysis" ) OR INDEXTERMS ( "diet, healthy" ) OR INDEXTERMS ( "economics, behavioral" ) OR INDEXTERMS ( "Body Mass Index" ) OR INDEXTERMS ( obesity ) OR INDEXTERMS ( overweight ) OR INDEXTERMS ( "Weight Loss" ) OR INDEXTERMS ( "Weight Gain" ) OR INDEXTERMS ( adiposity ) ) AND ( TITLE-ABS ( supermarket* ) OR TITLE-ABS ( "grocery store*" ) OR TITLE-ABS ( "food store*" ) OR TITLE-ABS ( grocer* ) OR TITLE-ABS ( hypermarket* ) OR TITLE-ABS ( bodega* ) OR TITLE-ABS ( "convenience store*" ) OR TITLE-ABS ( "food market" ) OR TITLE-ABS ( "food retail*" ) OR TITLE-ABS ( "retail food" ) OR ALL ( "retail store*" ) OR TITLE-ABS ( "food vendor*" ) OR INDEXTERMS ( supermarkets ) ) AND ( ( ( TITLE-ABS ( "Systematic Review" ) OR TITLE-ABS ( "scoping review" ) OR TITLE-ABS ( metanalysis ) OR TITLE-ABS ( metanalyses ) OR TITLE-ABS ( "meta analysis" ) OR TITLE-ABS ( meta-analyses ) OR ALL ( "narrative review" ) ) AND TITLE-ABS ( "literature review" ) ) OR TITLE-ABS ( "rapid review" ) OR TITLE-ABS ( "critically appraised topic" ) OR TITLE-ABS ( overview ) OR TITLE-ABS ( "collaborative review" ) OR TITLE-ABS ( "umbrella review" ) OR TITLE-ABS ( handsearch ) OR TITLE-ABS ( "hand search" ) OR TITLE-ABS ( "data synthesis" ) OR TITLE-ABS ( "data extraction" ) OR DOCTYPE ( "meta analysis" ) OR DOCTYPE ( review ) OR DOCTYPE ( "Systematic Review" ) OR INDEXTERMS ( "Meta-Analysis as Topic" ) OR INDEXTERMS ( "Review Literature as Topic" ) OR INDEXTERMS ( "Systematic Reviews as Topic" ) OR INDEXTERMS ( "Systematic Reviews as Topic" ) ) |  |
| **EconLit**  **[04.04.2023]** | (TI,AB("store tour*") OR TI,AB(intervention*) OR TI,AB("choice architecture") OR TI,AB(promotion) OR TI,AB(incentive*) OR TI,AB(disincentive*) OR TI,AB(priming) OR TI,AB(discount*) OR TI,AB(price) OR TI,AB(pricing) OR TI,AB(voucher) OR TI,AB(bonus) OR TI,AB(reward*) OR TI,AB(coupon*) OR TI,AB(token*) OR TI,AB(rebate*) OR TI,AB(refund*) OR TI,AB(reimburse*) OR TI,AB(display) OR TI,AB(remov*) OR TI,AB(layout) OR TI,AB(strateg*) OR TI,AB(advert*) OR TI,AB(market) OR TI,AB(Marketing) OR TI,AB(initiative*) OR TI,AB(program*) OR TI,AB("loyalty card*") OR TI,AB("point of purchase") OR TI,AB("point of sale") OR TI,AB("point of choice") OR TI,AB("product placement") OR TI,AB("food placement") OR TI,AB("shelf placement") OR TI,AB(proximity) OR TI,AB(positioning) OR TI,AB("retail environment*") OR TI,AB("food environment") OR TI,AB("product swap”) OR TI,AB("product signage") OR TI,AB("product availability") OR TI,AB("store layout") OR TI,AB("checkout area") OR TI,AB("store entrance") OR TI,AB("floor display") OR TI,AB("product recommendation") OR TI,AB(Advertising) OR TI,AB(advertisement)) AND (TI,AB(food*) OR TI,AB(groceries) OR TI,AB(snack*) OR TI,AB(fruit*) OR TI,AB(vegetable*) OR TI,AB(nuts) OR TI,AB(beverage*) OR TI,AB(legumes) OR TI,AB(drink) OR TI,AB(drinks) OR TI,AB(nutrition)) AND (TI,AB("dietary outcome") OR TI,AB("dietary-related outcome") OR TI,AB("health benefit") OR TI,AB("dietary intake") OR TI,AB("diet quality") OR TI,AB("eating behaviour*") OR TI,AB("eating behavior*") OR TI,AB("healthy eating") OR TI,AB("healthy diet") OR TI,AB(Healthy) OR TI,AB(healthier) OR TI,AB(healthiness) OR TI,AB(unhealthy) OR TI,AB(low-fat) OR TI,AB(low-sodium) OR TI,AB(low-sugar) OR TI,AB(low-fat) OR TI,AB(low-sodium) OR TI,AB(low-sugar) OR TI,AB(purchas*) OR TI,AB(consumption) OR TI,AB("sales data") OR TI,AB("sales outcome*") OR TI,AB("customer satisfaction") OR TI,AB("sales effect") OR TI,AB("sales effects") OR TI,AB("business outcome*") OR TI,AB(revenue) OR TI,AB(profitability) OR TI,AB(profit) OR TI,AB(margin) OR TI,AB(turnover) OR TI,AB(ROI) OR TI,AB(cost-benefit) OR TI,AB(cost-benefit) OR TI,AB("return on investment") OR TI,AB("item sales") OR TI,AB("total sales") OR TI,AB("commercial viability") OR TI,AB(opportunity-cost) OR TI,AB(opportunity-cost) OR TI,AB(value-proposition) OR TI,AB(value-proposition) OR TI,AB("foot traffic") OR TI,AB(patronage) OR TI,AB("customer loyalty") OR TI,AB("store loyalty") OR TI,AB("return customer") OR TI,AB("return customers") OR TI,AB("customer demand") OR TI,AB(BMI) OR TI,AB(bodyweight) OR TI,AB("body weight") OR TI,AB(Overweight) OR TI,AB("muscle mass") OR TI,AB("lean mass") OR TI,AB(Obesity) OR TI,AB("Weight Gain") OR TI,AB("Weight Loss") OR TI,AB(Adiposity)) AND (TI,AB(supermarket*) OR TI,AB("grocery store*") OR TI,AB("food store*") OR TI,AB(grocer*) OR TI,AB(hypermarket*) OR TI,AB(bodega*) OR TI,AB("convenience store*") OR TI,AB("food market") OR TI,AB("food retail*") OR TI,AB("retail food") OR NOFT("retail store*") OR TI,AB("food vendor*")) AND (((TI,AB("Systematic Review") OR TI,AB("scoping review") OR TI,AB(metanalysis) OR TI,AB(metanalyses) OR TI,AB("meta analysis") OR TI,AB(meta-analyses) OR NOFT("narrative review")) AND TI,AB("literature review")) OR TI,AB("rapid review") OR TI,AB("critically appraised topic") OR TI,AB(overview) OR TI,AB("collaborative review") OR TI,AB("umbrella review") OR TI,AB(handsearch) OR TI,AB("hand search") OR TI,AB("data synthesis") OR TI,AB("data extraction") OR NOFT("meta analysis") OR NOFT(Review) OR NOFT("Systematic Review")) |  |
| **Cochrane Central**  **[04.04.2023]** | (“store tour” OR “store tours” OR intervention* OR “choice architecture” OR promotion OR incentive* OR disincentive* OR priming OR discount* OR price OR pricing OR voucher OR bonus OR reward* OR coupon* OR token* OR rebate* OR refund* OR reimburse* OR display OR remov* OR layout OR strateg* OR advert* OR market OR marketing OR initiative* OR program* OR “loyalty card” OR “loyalty cards” OR “point of purchase” OR “point of sale” OR “point of choice” OR “product placement” OR “food placement” OR “shelf placement” OR proximity OR positioning OR “retail environment” OR “retail environments” OR “food environment” OR “product swap” OR “product signage” OR “product availability” OR “store layout” OR “checkout area” OR “store entrance” OR “floor display” OR “product recommendation” OR advertising OR advertisement):ti,ab AND (Food* OR groceries OR snack* OR fruit* OR vegetable* OR nuts OR beverage* OR legumes OR drink OR drinks OR nutrition):ti,ab AND (“dietary outcome” OR “dietary-related outcome” OR “health benefit” OR “dietary intake” OR “diet quality” OR (eating NEXT behaviour*) OR (eating NEXT behavior*) OR “healthy eating” OR “healthy diet” OR Healthy OR healthier OR healthiness OR unhealthy OR “low fat” OR “low sodium” OR “low sugar” OR low-fat OR low-sodium OR low-sugar OR purchas* OR consumption OR “sales data” OR (sales NEXT outcome*) OR “customer satisfaction” OR “sales effect*” OR (business NEXT outcome*) OR revenue OR profitability OR profit OR margin OR turnover OR ROI OR cost-benefit OR “cost benefit” OR “return on investment” OR “item sales” OR “total sales” OR “commercial viability” OR “opportunity cost” OR opportunity-cost OR value-proposition OR “value proposition” OR “foot traffic” OR patronage OR “customer loyalty” OR “store loyalty” OR (return NEXT customer*) OR “customer demand” OR BMI OR bodyweight OR “body weight” OR overweight OR “muscle mass” OR “lean mass” OR obesity OR “weight gain” OR “weight loss” OR adiposity):ti,ab AND (Supermarket* OR (grocery NEXT store*) OR (food NEXT store*) OR grocer* OR hypermarket* OR bodega* OR (convenience NEXT store*) OR (food NEXT market) OR (food NEXT retail*) OR”retail food”):ti,ab AND (“systematic review” OR “scoping review” OR metanalysis OR metanalyses OR meta-analysis OR meta-analyses OR “narrative review” OR “literature review” OR “rapid review” OR “critically appraised topic” OR overview OR “collaborative review” OR “umbrella review” OR handsearch OR “hand search” OR “data synthesis” OR “data extraction”):ti,ab" | reviews only |
| **Google Scholar**  **[04.04.2023]** | (intervention OR “food environment” OR “retail environment”) AND (food OR fruit OR vegetable* OR drink*) AND (diet* OR health* OR sale* OR purchase* OR ROI OR cost* OR profit*) AND (supermarket* OR "grocery store") AND ("systematic review" OR "scoping review" OR “data extraction” OR overview OR (umbrella OR rapid OR literature) AND review) | First 200 records |
| **ProQuest**  **[04.04.2023]** | (TI,AB("store tour*") OR TI,AB(intervention*) OR TI,AB("choice architecture") OR TI,AB(promotion) OR TI,AB(incentive*) OR TI,AB(disincentive*) OR TI,AB(priming) OR TI,AB(discount*) OR TI,AB(price) OR TI,AB(pricing) OR TI,AB(voucher) OR TI,AB(bonus) OR TI,AB(reward*) OR TI,AB(coupon*) OR TI,AB(token*) OR TI,AB(rebate*) OR TI,AB(refund*) OR TI,AB(reimburse*) OR TI,AB(display) OR TI,AB(remov*) OR TI,AB(layout) OR TI,AB(strateg*) OR TI,AB(advert*) OR TI,AB(market) OR TI,AB(Marketing) OR TI,AB(initiative*) OR TI,AB(program*) OR TI,AB("loyalty card*") OR TI,AB("point of purchase") OR TI,AB("point of sale") OR TI,AB("point of choice") OR TI,AB("product placement") OR TI,AB("food placement") OR TI,AB("shelf placement") OR TI,AB(proximity) OR TI,AB(positioning) OR TI,AB("retail environment*") OR TI,AB("food environment") OR TI,AB("product swap”) OR TI,AB("product signage") OR TI,AB("product availability") OR TI,AB("store layout") OR TI,AB("checkout area") OR TI,AB("store entrance") OR TI,AB("floor display") OR TI,AB("product recommendation") OR TI,AB(Advertising) OR TI,AB(advertisement)) AND (TI,AB(food*) OR TI,AB(groceries) OR TI,AB(snack*) OR TI,AB(fruit*) OR TI,AB(vegetable*) OR TI,AB(nuts) OR TI,AB(beverage*) OR TI,AB(legumes) OR TI,AB(drink) OR TI,AB(drinks) OR TI,AB(nutrition)) AND (TI,AB("dietary outcome") OR TI,AB("dietary-related outcome") OR TI,AB("health benefit") OR TI,AB("dietary intake") OR TI,AB("diet quality") OR TI,AB("eating behaviour*") OR TI,AB("eating behavior*") OR TI,AB("healthy eating") OR TI,AB("healthy diet") OR TI,AB(Healthy) OR TI,AB(healthier) OR TI,AB(healthiness) OR TI,AB(unhealthy) OR TI,AB(low-fat) OR TI,AB(low-sodium) OR TI,AB(low-sugar) OR TI,AB(low-fat) OR TI,AB(low-sodium) OR TI,AB(low-sugar) OR TI,AB(purchas*) OR TI,AB(consumption) OR TI,AB("sales data") OR TI,AB("sales outcome*") OR TI,AB("customer satisfaction") OR TI,AB("sales effect") OR TI,AB("sales effects") OR TI,AB("business outcome*") OR TI,AB(revenue) OR TI,AB(profitability) OR TI,AB(profit) OR TI,AB(margin) OR TI,AB(turnover) OR TI,AB(ROI) OR TI,AB(cost-benefit) OR TI,AB(cost-benefit) OR TI,AB("return on investment") OR TI,AB("item sales") OR TI,AB("total sales") OR TI,AB("commercial viability") OR TI,AB(opportunity-cost) OR TI,AB(opportunity-cost) OR TI,AB(value-proposition) OR TI,AB(value-proposition) OR TI,AB("foot traffic") OR TI,AB(patronage) OR TI,AB("customer loyalty") OR TI,AB("store loyalty") OR TI,AB("return customer") OR TI,AB("return customers") OR TI,AB("customer demand") OR TI,AB(BMI) OR TI,AB(bodyweight) OR TI,AB("body weight") OR TI,AB(Overweight) OR TI,AB("muscle mass") OR TI,AB("lean mass") OR TI,AB(Obesity) OR TI,AB("Weight Gain") OR TI,AB("Weight Loss") OR TI,AB(Adiposity)) AND (TI,AB(supermarket*) OR TI,AB("grocery store*") OR TI,AB("food store*") OR TI,AB(grocer*) OR TI,AB(hypermarket*) OR TI,AB(bodega*) OR TI,AB("convenience store*") OR TI,AB("food market") OR TI,AB("food retail*") OR TI,AB("retail food") OR NOFT("retail store*") OR TI,AB("food vendor*")) AND (((TI,AB("Systematic Review") OR TI,AB("scoping review") OR TI,AB(metanalysis) OR TI,AB(metanalyses) OR TI,AB("meta analysis") OR TI,AB(meta-analyses) OR NOFT("narrative review")) AND TI,AB("literature review")) OR TI,AB("rapid review") OR TI,AB("critically appraised topic") OR TI,AB(overview) OR TI,AB("collaborative review") OR TI,AB("umbrella review") OR TI,AB(handsearch) OR TI,AB("hand search") OR TI,AB("data synthesis") OR TI,AB("data extraction")) |  |

*Note:* Date format, dd.mm.yyy.

**Appendix C: Explanatory notes and quality appraisal of included reviews (Part I)**

Table S2. Quality appraisal of reviews using the Risk of Bias In Systematic Reviews (ROBIS) tool

|  | **Phase 1** | | | | | | | | | | | | | | | | | | | | | | | | | **Phase 2** | | | **Overall risk of bias** |  |
| --- | --- | --- | --- | --- | --- | --- | --- | --- | --- | --- | --- | --- | --- | --- | --- | --- | --- | --- | --- | --- | --- | --- | --- | --- | --- | --- | --- | --- | --- | --- |
|  | **Study eligibility criteria** | | | | | | **Identification and selection of studies** | | | | | | **Data collection and study appraisal** | | | | | | **Synthesis and findings** | | | | | | | **Conclusions supported by evidence** | | |  |  |
| **Author, year** | **1.1** | **1.2** | **1.3** | **1.4** | **1.5** | **Overall** | **2.1** | **2.2** | **2.3** | **2.4** | **2.5** | **Overall** | **3.1** | **3.2** | **3.3** | **3.4** | **3.5** | **Overall** | **4.1** | **4.2** | **4.3** | **4.4** | **4.5** | **4.6** | **Overall** | **A** | **B** | **C** |  |  |
| **Adam & Jensen 2016 (4)** | PY | Y | Y | N | Y | **Low concern** | PY | Y | PY | PY | N | **Low concern** | NI | PY | PY | Y | NI | **High concern** | PY | NI | PY | N | PY | Y | **Low concern** | PY | Y | Y | **Low risk of bias** |  |
| **Afshin et al. 2017 (5)** | Y | Y | Y | N | Y | **Low concern** | PY | Y | Y | Y | NI | **Low concern** | Y | Y | PY | PY | Y | **Low concern** | Y | PY | PY | Y | PY | Y | **Low concern** | Y | Y | PY | **Low risk of bias** |  |
| **Alston et al. 2020 (6)** | PY | Y | Y | N | PY | **Low concern** | PY | N | PY | N | Y | **High concern** | Y | PY | PY | Y | Y | **Low concern** | PY | NI | PY | N | PY | Y | **Low concern** | PY | Y | PY | **Low risk of bias** |  |
| **An 2013 (7)** | PY | Y | Y | N | PY | **Low concern** | PY | Y | PY | N | NI | **High concern** | N | PY | PY | PY | NI | **Unclear concern** | PY | NI | PY | N | PY | N | **High concern** | N | PY | PY | **Unclear risk of bias** |  |
| **Blake et al. 2019 (3)** | Y | Y | Y | N | Y | **Low concern** | Y | Y | PY | PY | PY | **Low concern** | Y | Y | PY | Y | Y | **Low concern** | PY | Y | PY | Y | PY | N | **Low concern** | Y | Y | PY | **Low risk of bias** |  |
|  |  |  |  |  |  |  |  |  |  |  |  |  |  |  |  |  |  |  |  |  |  |  |  |  |  |  |  |  |  |  |
| **Cameron et al. 2016 (8)** | PY | Y | Y | N | Y | **Low concern** | Y | Y | PN | PY | N | **High concern** | NI | PY | PY | Y | Y | **Low concern** | Y | NI | PY | Y | PY | Y | **Low concern** | PY | Y | Y | **Low risk of bias** |  |
| **Chan et al. 2021 (9)** | PY | Y | Y | N | PY | **Low concern** | PY | PY | Y | N | PY | **Low concern** | N | Y | PY | Y | NI | **Unclear concern** | PY | NI | PY | N | PY | N | **High concern** | PN | Y | PY | **Unclear risk of bias** |  |
| **Crockett et al. 2018 (10)** | Y | Y | Y | Y | Y | **Low concern** | Y | Y | Y | Y | Y | **Low concern** | Y | Y | Y | Y | NI | **Low concern** | Y | Y | Y | Y | PY | Y | **Low concern** | Y | Y | Y | **Low risk of bias** |  |
| **Epstein et al. 2012 (11)** | PY | Y | Y | N | PY | **Low concern** | PY | N | PN | N | NI | **High concern** | NI | PY | PY | N | N | **High concern** | PY | NI | PY | N | PY | N | **High concern** | N | PY | PY | **High risk of bias** |  |
| **Escaron et al. 2013 (12)** | PY | Y | Y | N | Y | **Low concern** | PN | N | PN | N | N | **High concern** | N | PY | PY | PY | N | **High concern** | PY | NI | PY | N | PY | Y | **Low concern** | N | Y | PY | **Unclear risk of bias** |  |
| **Fergus et al. 2021 (13)** | Y | Y | Y | N | PY | **Low concern** | Y | Y | PN | N | N | **High concern** | N | Y | PY | Y | Y | **Low concern** | PY | PY | PY | N | PY | Y | **Low concern** | PY | Y | PY | **Low risk of bias** |  |
| **Gittelsohn et al. 2017 (14)** | PY | Y | Y | N | PY | **Low concern** | PY | Y | Y | N | Y | **Low concern** | Y | Y | PY | PY | NI | **Low concern** | PY | NI | PY | Y | PY | N | **Low concern** | Y | Y | PY | **Low risk of bias** |  |
| **Glanz et al. 2012 (15)** | PY | Y | Y | N | Y | **Low concern** | PY | Y | PN | PY | NI | **High concern** | NI | PY | PY | N | N | **High concern** | NI | NI | PY | N | PY | N | **High concern** | N | PY | N | **High risk of bias** |  |
| **Golding et al. 2022 (16)** | Y | Y | Y | N | Y | **Low concern** | PY | Y | Y | PY | N | **Low concern** | Y | Y | PY | Y | Y | **Low concern** | PY | Y | PY | N | PY | Y | **Low concern** | Y | Y | PY | **Low risk of bias** |  |
| **Harbers et al. 2020 (17)** | Y | Y | Y | N | PY | **Low concern** | PY | Y | Y | N | Y | **Low concern** | N | Y | PY | Y | Y | **Low concern** | PY | Y | PY | Y | PY | Y | **Low concern** | Y | Y | Y | **Low risk of bias** |  |
| **Hartmann-Boyce et al. 2018 (18)** | Y | Y | Y | N | Y | **Low concern** | PY | Y | PY | NI | Y | **Low concern** | Y | Y | PY | Y | Y | **Low concern** | Y | Y | PY | Y | PY | N | **Low concern** | Y | Y | Y | **Low risk of bias** |  |
| **Hodges et al. 2023 (19)** | Y | Y | Y | N | Y | **Low concern** | Y | PY | Y | PY | Y | **Low concern** | Y | PY | PY | PY | NI | **Low concern** | PY | PY | PY | N | PY | N | **High concern** | PY | Y | PY | **Low risk of bias** |  |
| **Hollands et al. 2019 (20)** | PY | Y | Y | Y | Y | **Low concern** | Y | Y | Y | Y | Y | **Low concern** | Y | Y | Y |  | Y | **Low concern** | Y | Y | Y | Y | PY | Y | **Low concern** | Y | Y | Y | **Low risk of bias** |  |
|  |  |  |  |  |  |  |  |  |  |  |  |  |  |  |  | Y |  |  |  |  |  |  |  |  |  |  |  |  |  |  |
| **Karpyn et al. 2020 (21)** | PY | Y | Y | N | Y | **Low concern** | PY | Y | PY | N | Y | **Low concern** | Y | Y | PY | PY | NI | **Low concern** | PY | NI | PY | N | PY | N | **High concern** | PY | Y | PY | **Low risk of bias** |  |
| **Liberato et al. 2014 (22)** | PY | Y | Y | N | Y | **Low concern** | PY | PY | PY | Y | PY | **Low concern** | Y | Y | PY | Y | Y | **Low concern** | Y | NI | PY | Y | PY | N | **Low concern** | Y | Y | Y | **Low risk of bias** |  |
| **Mah et al. 2019 (23)** | PY | Y | Y | N | Y | **Low concern** | PY | Y | PY | N | Y | **Low concern** | N | PY | PY | N | N | **High concern** | Y | NI | PY | Y | PY | N | **Low concern** | PY | Y | PY | **Low risk of bias** |  |
| **Nikniaz et al. 2020 (24)** | PY | Y | Y | N | PY | **Low concern** | Y | Y | PY | N | Y | **Low concern** | Y | Y | PY | Y | NI | **Low concern** | PY | NI | PY | N | PY | N | **High concern** | PY | Y | PY | **Low risk of bias** |  |
| **Nikolaus et al. 2016 (25)** | PY | Y | Y | N | PY | **Low concern** | PY | Y | PN | N | N | **High concern** | Y | Y | PY | Y | Y | **Low concern** | PY | NI | PY | N | PY | Y | **Low concern** | PY | Y | PY | **Low risk of bias** |  |
| **Seymour et al. 2004 (26)** | PY | Y | Y | N | PY | **Low concern** | PY | Y | PN | N | NI | **High concern** | NI | Y | PY | PY | Y | **Low concern** | PY | NI | PY | N | PY | N | **High concern** | N | Y | PY | **Unclear of bias** |  |
| **Shangguan et al. 2019 (27)** | PY | Y | Y | Y | PY | **Low concern** | PY | Y | Y | Y | N | **Low concern** | Y | Y | PY | Y | Y | **Low concern** | Y | PY | PY | Y | PY | N | **Low concern** | Y | Y | PY | **Low risk of bias** |  |
| **Shaw et al. 2020 (28)** | PY | Y | Y | N | Y | **Low concern** | Y | Y | Y | PY | N | **Low concern** | PY | Y | PY | Y | Y | **Low concern** | PY | PY | PY | Y | PY | N | **Low concern** | Y | Y | PY | **Low risk of bias** |  |
| **Slapø et al. 2021 (29)** | PY | Y | Y | Y | Y | **Low concern** | PY | Y | PY | N | Y | **Low concern** | NI | Y | PY | Y | Y | **Low concern** | Y | PY | PY | Y | PY | Y | **Low concern** | Y | Y | Y | **Low risk of bias** |  |
| **Valencic et al. 2022 (30)** | PY | Y | Y | N | Y | **Low concern** | PY | Y | PY | N | PY | **Low concern** | Y | Y | PY | N | N | **High concern** | Y | NI | PY | Y | PY | N | **Low concern** | PY | PY | PY | **Low risk of bias** |  |
| **van’tRiet 2013 (31)** | PY | Y | Y | N | PY | **Low concern** | PY | Y | PN | PY | N | **High concern** | N | PY | Y | N | N | **High concern** | PY | NI | PY |  | PY | N | **Low concern** | N | Y | PY | **Unclear risk of bias** |  |
|  |  |  |  |  |  |  |  |  |  |  |  |  |  |  |  |  |  |  |  |  |  | Y |  |  |  |  |  |  |  |  |
| **Vecchio et al. 2019 (32)** | N | N | N | N | PY | **High concern** | PY | Y | PN | N | Y | **High concern** | NI | Y | PY | Y | Y | **Low concern** | PY | NI | PY | N | PY | N | **High concern** | N | Y | PY | **High risk of bias** |  |
| **von Philipsborn et al. 2019 (33)** | PY | Y | Y | Y | Y | **Low concern** | Y | Y | Y | Y | Y | **Low concern** | Y | Y | Y | Y | Y | **Low concern** | Y | Y | Y | Y | PY | Y | **Low concern** | Y | Y | Y | **Low risk of bias** |  |
|  |  |  |  |  |  |  |  |  |  |  |  |  |  |  |  |  |  |  |  |  |  |  |  |  |  |  |  |  |  |  |
| **Wolgast et al. 2022 (34)** | PY | Y | Y | N | PY | **Low concern** | PY | Y | PY | PY | Y | **Low concern** | Y | PY | PY | N | N | **High concern** | PY | NI | PY | N | PY | N | **High concern** | N | Y | PY | **Unclear risk of bias** |  |
| **Wyse et al. 2021 (35)** | Y | Y | Y | N | Y | **Low concern** | Y | Y | PY | Y | Y | **Low concern** | Y | Y | Y | Y | Y | **Low concern** | PY | PY | PY | Y | PY | Y | **Low concern** | Y | Y | Y | **Low risk of bias** |  |
| **Yoder et al. 2021 (36)** | NI | Y | Y | N | Y | **Low concern** | Y | Y | PY | N | NI | **High concern** | NI | N | PY | N | N | **High concern** | PY | NI | PY | N | PY | N | **High concern** | N | PY | PY | **High risk of bias** |  |

*Note: Studies deemed as ‘low risk of bias’ are considered ‘high quality’, and studies deemed ‘high risk of bias’ are considered ‘low quality’.*

*Detailed explanatory notes about the ROBIS tool:* **Phase 1. Domain 1, Study eligibility criteria**: 1.1 Did the review adhere to pre-defined objectives and eligibility criteria?; 1.2 Were the eligibility criteria appropriate for the review question?; 1.3 Were eligibility criteria unambiguous?; 1.4 Were any restrictions in eligibility criteria based on study characteristics appropriate?; 1.5 Were any restrictions in eligibility criteria based on sources of information appropriate (e.g. publication status or format, language, availability of data?. **Domain 2, Identification and selection of studies**: 2.1 Did the search include an appropriate range of databases/electronic sources for published and unpublished reports?; 2.2 Were methods additional to database searching used to identify relevant reports?; 2.3 Were the terms and structure of the search strategy likely to retrieve as many eligible studies as possible?; 2.4 Were restrictions based on date, publication format, or language appropriate?; 2.5 Were efforts made to minimise error in selection of studies?. **Domain 3, Data collection and study appraisal**: 3.1 Were efforts made to minimise error in data collection?; 3.2 Were sufficient study characteristics available for both review authors and readers to be able to interpret the results?; 3.3 Were all relevant study results collected for use in the synthesis?; 3.4 Were efforts made to minimise error in risk of bias assessment?; 3.5 Was risk of bias (or methodological quality) formally assessed using appropriate criteria?. **Domain 4, Synthesis and findings**: 4.1 Did the synthesis include all studies that it should?; 4.2 Were all pre-defined analyses reported or departures explained?; 4.3 Was the synthesis appropriate given the nature and similarity in the research questions, study designs and outcomes across included studies?; 4.4 Was between-study variation (heterogeneity) minimal or addressed in the synthesis?; 4.5 Were the findings robust, e.g. as demonstrated through funnel plot or sensitivity analyses?; 4.6 Were biases in primary studies minimal or addressed in the synthesis?. **Phase 2. Domain 5, Describe whether conclusions were supported by the evidence:** 5A Did the interpretation of findings address all of the concerns identified in Domains 1 to 4?; 5B Was the relevance of identified studies to the review’s research question appropriately considered?; 5C Did the reviewers avoid emphasizing results on the basis of their statistical significance?.

Table S3. Quality appraisal of reviews using the Scale for the Assessment of Narrative Review Articles (SANRA) tool

| **Author, year** | **Domain 1** | **Domain 2** | **Domain 3** | **Domain 4** | **Domain 5** | **Domain 6** | **Total score (/12)** |
| --- | --- | --- | --- | --- | --- | --- | --- |
| **Cohen et al. 2012 (37)** | 2 | 2 | 0 | 2 | 1 | 1 | **8 [Unclear risk of bias]** |
| **Glanz & Yaroch 2004 (38)** | 1 | 2 | 0 | 2 | 1 | 1 | **7 [Unclear risk of bias]** |
| **Mayer et al. 1989 (39)** | 2 | 1 | 0 | 2 | 2 | 1 | **8 [Unclear risk of bias]** |
| **Volkova et al. 2015 (40)** | 2 | 2 | 0 | 2 | 2 | 2 | **10 [High quality]** |

*Detailed explanatory notes about the SANRA tool:* **Domain 1,** explanation of the review’s importance; **Domain 2,** statement of the aims of the review**; Domain 3,** description of the literature search**; Domain 4,** referencing; **Domain 5,** scientific reasoning; **Domain 6,** presentation of relevant and appropriate endpoint data. The six domains that form the scale are rated in integers from 0 (low standard) to 2 (high standard), with 1 as an intermediate score. The sum score of the scale is intended to measure the construct “quality of a narrative review article”; a score of 4 or below indicates very poor quality (41).

**Additional explanatory notes:**

For a review to be scored as ‘high quality’ overall, the critical domains (5, scientific reasoning; and 6, appropriate presentation of data) must have received a score of 2 (the maximum score) in each domain. The remaining four domains (1, justification of the article’s importance for the readership; 2, statement of concrete aims or formulation of questions; 3, description of the literature search; and 4, referencing) could include either (i) a maximum of one low standard score (0) rating, or (ii) up to two intermediate scores (1), but must not include any domains scored as low standard (0).

**Appendix D: Additional explanatory notes to describe the quality appraisal scoring system for primary studies where an overall rating was not provided by original review authors**

For a primary research article to be scored as ‘high quality’ on the Cochrane risk of bias tool (1), the two critical domains (selective reporting and incomplete outcome data) must have been rated as *low risk of bias* in each domain. All other domains could include either (i) no more than one *high risk of bias* rating, or (ii) no more than two *unclear risk of bias* ratings, but must not include any *high risk of bias* ratings. For a primary research article to be scored as ‘high quality’ on the Joanna Briggs Institute Critical Appraisal tool (2), it must have received an overall summary score of at least 75%, which was calculated by dividing the number of ‘yes’ responses by the total number of items. For a primary research article to be scored as ‘high quality’ on the Mixed Methods Appraisal Tool, as used by Blake et al. (3), it must have received an overall summary score of 100%.

**Appendix E: Characteristics and findings of reviews excluded from the overview of reviews synthesis on the basis of their quality appraisal**

Table S4. Characteristics of reviews excluded in the overview of reviews synthesis

| **Reference**  **[author, year]** | **Review type** | **Review eligibility criteria** | | | **Search strategy** | | **No. of included primary studies** | **Quality** |
| --- | --- | --- | --- | --- | --- | --- | --- | --- |
|  |  | **Research design** | **Population and setting** | **Intervention strategy** | **No. of databases** | **Search period** |  |  |
| **An, 2013 (7) ^^^** | Systematic review | RCTs, cohort, pre-post | Population: Adolescents (12-17 years) or adults (>18 years)  Setting: NR | Monetary subsides (i.e., price discounts or vouchers for healthier foods) | 5 | 1 Jan 1990 to 1 May 2012 | 24 | Unclear |
| **Chan et al., 2021 (9) ^^^** | Systematic review | RCTs and quasi-experimental trials | Population: Any  Setting: Food retail outlets (physical) | Point-of-sale nutrition information | 5 | 1 Jan 2000 to 3 Apr 2020 | 26 | Unclear |
| **Cohen et al., 2012 (37)** | Narrative literature review | NR | Population: NR  Setting: Restaurants and grocery stores | Superficial characteristics of food products: packaging and portion sizes, design, salience, health claims and labelling | NR | NR | Not indicated | Unclear |
| **Epstein et al. 2012 (11) ^^^** | Targeted review | Experimental studies | Population: NR  Setting: Laboratories, cafeterias, restaurants, vending machines, supermarkets, farmers’ markets (laboratory or field) | Price manipulation (i.e., subsidies, discounts) | 4 | Jan 1980 and Mar 2011 | 24 | Low quality |
| **Escaron et al., 2013 (12) ^^^** | Systematic review | NR | Population: NR  Setting: Supermarkets and grocery stores | Point of purchase information, pricing, increased availability of healthful foods, and promotion and advertising | 1 | NR (1978 to Jul 2012) ^§^ | 58 | Unclear |
| **Glanz & Yaroch, 2004 (38) ^*^** | Narrative literature review | NR | Population: NR  Setting: Grocery stores and community settings | Environmental, policy, and pricing initiatives | NR | NR | 35 | Unclear |
| **Glanz et al., 2012 (15) ^^^** | Integrative review | Controlled laboratory experiments, observation, and field experiments | Population: NR  Setting: Grocery stores (physical or simulated) | Marketing influences on food purchases or consumption (i.e., 4Ps: product, price, place, and promotion) | 8 + grey literature searched | 1995 to 2010 | 125 | Low quality |
| **Mayer et al., 1989 (39) ^*^** | Narrative literature review | Controlled evaluations | Population: NR  Setting: Restaurants, cafeterias, and supermarkets | Point-of-choice interventions | NR | NR | 13 | Unclear |
| **Seymour et al., 2004 (26) ^^^** | Systematic review | NR | Population: Adult population  Setting: Worksites, universities, grocery stores and restaurants | Environmental (availability, access, incentives, or information about foods at the point of purchase) and policy nutrition interventions | 5 | 1970 to June 2003 | 38 | Unclear |
| **van'tRiet, 2013 (31) ^^^** | Systematic review | Experimental design | Population: Supermarket clientele   Setting: Supermarkets or grocery stores | Product health information at point-of-purchase | 4 | 1980 to Jan 2010 | 16 | Unclear |
| **Vecchio et al., 2019 (32) ^*^** | Systematic review | NR | Population: NR  Setting: NR | Nudging approaches (i.e., altering the physical environment) | 2 | 2015 to 2018 | 26 | Low quality |
| **Wolgast et al., 2022 (34) ^^^** | Systematic review | Intervention, pilot, experimental studies | Population: Customers  Setting: Supermarket, grocery store, corner store, bodega, retail environment (physical) | In-store marketing interventions for healthy food and beverage promotion | 9 | 1 Jan 2010 to 6 Jun 2022 | 73 | Unclear |
| **Yoder et al., 2021 (36) ^^^** | Scoping review | RCTs, non-RCTs, or observational studies | Population: General population >18 years or organisations  Setting: Food retail stores: grocery stores, supermarkets, corner stores, convenience stores (online or physical) | Increase awareness, knowledge, and/or skills of food purchasing decisions; impacted consumer demand, accessibility, and/or affordability to choose healthier foods and drinks | 8 + grey literature searched | 1970 to 2 Oct 2019 | 211 | Low quality |

*Note*: ^^^, indicates study quality was assessed using the ROBIS tool; ^*^, indicates study quality was assessed using the SANRA tool; ^§^ , extrapolated from results.

*Abbreviations*: NR, not reported; RCT, randomised controlled trial.

Table S5. Findings of reviews excluded in the overview of reviews synthesis

| **Reference**  **[author, year]** | **Settings** | **Outcomes** | **Overview of findings** | **Tool used to assess quality of primary studies** |
| --- | --- | --- | --- | --- |
| **An, 2013 (7)** | **Mixed:** Supermarkets, cafeterias, vending machines, farmers’ markets, restaurants, and organic food stores | **Primary outcome(s):** Food purchases or consumption | - Improved affordability was associated with significant increases in the purchase and consumption of healthier foods | Following Wu et al. (2011), the quality of each study included in the review was assessed by the presence or absence of 10 dichotomous criteria |
| **Chan et al., 2021 (9)** | **Mixed:** Supermarkets, grocery stores, community stores, corner stores, conveniences stores, and discount food stores | **Primary outcome(s):** Healthier food purchases and consumption | - Point-of-sale interventions identifying healthy and unhealthy options can result in healthier customer purchasing/intake behaviour, with particularly promising evidence for shelf-label nutrition summary systems and technology delivered strategies. In-store displays had mixed effectiveness. | Effective Public Health Practice Project Quality Assessment Tool for Quantitative Studies |
| **Cohen et al., 2012 (37)** | **Mixed:** Restaurants and grocery stores/supermarkets | **Primary outcome(s):** Food choice and consumption | - In both settings, attempts to help people make better choices by pointing out healthful characteristics do not seem to be sufficient to overcome the automatic responses to the promotion of less healthful items. In restaurants, the strongest factors appear to be the presentation of food choices and the quantity served. In supermarkets, saliency and presentation are key factors that influence choice. | N/A |
| **Epstein et al. 2012 (11)** | **Mixed:** Laboratory settings, cafeterias, restaurants, vending machines, supermarkets and farmer’s markets | **Primary outcome(s):** Purchases of targeted and nontargeted foods, total energy, or macronutrients purchased | - Price changes influence food-purchasing patterns. Increasing prices of less healthy foods reduced their purchase and reducing prices of healthier options increased their purchase. - Experimental research suggests that price changes modify purchases of targeted foods, but research on the overall nutritional quality of purchases is mixed because of substitution effects. There is mixed support for combining price changes with adjunctive interventions, and there are no replicated findings on moderators to price sensitivity in experiments. | N/A |
| **Escaron et al., 2013 (12)** | Grocery stores and supermarkets | **Primary outcome(s):** Awareness and use of intervention materials, sales data, customers’ knowledge and beliefs, preferences, intentions, and process measures.   Where reported, F&V, fat or dietary intake were extracted | - Interventions combining demand as well as supply-side strategies have sufficient evidence to influence customers and management toward more healthful food purchases | Suitability of the study design to determine effectiveness, scored as 1, 2, or 3 points |
| **Glanz & Yaroch, 2004 (38)** | **Mixed:** Grocery stores, supermarkets, convenience stores, farmer’s markets, snack shops, virtual grocery, churches, child care centres, neighbourhoods and multisectoral community programs | **Primary outcome(s):** Fruit and vegetable intake | - Four key types of grocery-store-based interventions include point-of-purchase (POP) information; reduced prices and coupons; increased availability, variety, and convenience; and promotion and advertising. There is strong support for the feasibility of these approaches and modest evidence of their efficacy in influencing eating behaviour. | N/A |
| **Glanz et al., 2012 (15)** | Grocery stores | **Primary outcome(s):** Food purchases or consumption | - Findings suggest several strategies for in-store marketing to promote healthful eating by increasing availability, affordability, prominence, and promotion of healthful foods and/or restricting or de-marketing unhealthy foods. | N/A |
| **Mayer et al., 1989 (39)** | **Mixed:** Cafeterias, restaurants, fast-food restaurants, and supermarkets | **Primary outcome(s):** Behaviour change | - All programs included either nutrient information alone or nutrient information combined with an incentive strategy - In three of the five studies, point-of-choice interventions in supermarkets produced either no changes in food purchase behaviour or patterns of changes that were difficult to interpret - Labelling strategies have been effective in promoting more nutritious food selections in dining establishments | N/A |
| **Seymour et al., 2004 (26)** | **Mixed:** Worksites (cafeterias and vending machines), universities, grocery stores, restaurants | **Primary outcome(s):** Change in dietary patterns, sales of targeted foods, food choices | - Many interventions were not thoroughly evaluated or lacked important evaluation information. Direct comparison of studies across settings was not possible, but available data suggest that worksite and university interventions have the most potential for success. Interventions in grocery stores appear to be the least effective. | Each study was given a rating from one to four asterisks that was based on the quality of the research design (developed from Ammerman et al. (2002) and Wilson (1996)). |
| **van'tRiet, 2013 (31)** | Supermarkets and grocery stores | **Primary outcome(s):** Purchase behaviour (sales data provided by stores or from customer receipts) | - Evidence for the effectiveness of product health information is mixed. It appears that interventions are more successful when they are implemented for longer than a year, when they are accompanied by additional promotional activities and when they target the absence of unhealthy nutrients, such as fat, sugar, or calories, instead of or in addition to the presence of healthy nutrients, such as minerals, vitamins, or fibre. | N/A |
| **Vecchio et al., 2019 (32)** | **Mixed:** Grocery stores, supermarkets, corner stores, schools | **Primary outcome(s):** Healthy food choice | - Nudging approaches were effective, with over 80% of the reviewed research reporting positive outcomes | Assessed using twenty items (resulting in 21 points) of the checklist proposed by Downs and Black (1998). |
| **Wolgast et al., 2022 (34)** | **Mixed:** Supermarkets, bodega or corner store, low-income community, SNAP enrolees | **Primary outcome(s):** Purchase-related (i.e., objective store sales data, objective food purchasing data, customer receipts, and survey self-reported purchases or expenditures, store sales, or intent to purchase), and/or consumption-related (i.e., food frequency questionnaires (FFQ), 24 h dietary recalls, food diary, Veggie Meter or other biometrics, or other self-reported diet/consumption or intent to eat surveys) | - The following unique intervention types were considered as evaluated in these studies for their ability to increase healthy item purchasing and consumption: (1) nutrition scoring, (2) nutritional messaging, (3) non-nutritional messaging, (4) endcaps and secondary placement, (5) point-of-sale interventions, (6) increased stocking, (7) food tasting and demonstrations, (8) nutrition education, and (9) placement on shelf interventions. - Nutritional scoring and nutritional messaging emerged as the most rigorously tested and effective intervention strategies. Other strategies warrant more research attention. Simple intervention strategies, as opposed to complex ones, yield the most successful results and minimize shopper burden. | N/A |
| **Yoder et al., 2021 (36)** | **Mixed:** Grocery stores, supermarkets, farmer’s markets/mobile produce markets, multiple settings, such as grocery stores, farmer’s markets, and other retail locations such as drug store/pharmacies, corner/convenience stores, online retail settings and retail clinics | **Primary outcome(s):** Improvement in health outcomes (nutritional quality of shopping purchases, eating habits, biometric measures) and cost effectiveness (store sales, health care dollar saving) | - The literature indicates utilizations of multiple intervention categories, such as incentive programs, personalized nutrition education, and path-to-purchase marketing, will produce both health outcomes and improved return on investment for the food retailer | N/A |

*Note*: F&V, fruit and vegetables; N/A, not applicable; SNAP, Supplemental Nutrition Assistance Program; 4Ps, Price, Product, Place, Promotion.

**Appendix F: Findings of high-quality reviews included in the overview of reviews**

Table S6. Effects of interventions, synthesised by high-quality included reviews

| **Reference**  **[author, year]** | **Settings** | **Intervention strategy**^#^ | **Outcomes** | **Overview of findings** | **Tool used to assess quality of primary studies** |
| --- | --- | --- | --- | --- | --- |
| **Adam & Jensen, 2016 (4)** | **Mixed:** Supermarkets, worksite cafeterias, grocery stores, convenience stores, corner stores, farmers’ market | Pricing, prompting, product availability, combined | **Primary outcome(s):** Purchase of healthy foods   **Secondary outcome(s):** Consumption of healthy foods | - Price interventions with enough discount depth are promising, especially when combined with other strategies - Most interventions used a combination of information (e.g., awareness raising through food labelling, promotions, campaigns, etc.) and making healthy food available for consumers. - Price incentives contribute significantly to the effectiveness of intervention strategies, especially when combined with other components such as nutrition knowledge. | Risk of bias in line with Cochrane guidelines and Preferred Reporting Items for Systematic Reviews and Meta-Analyses (PRISMA). |
| **Afshin et al., 2017 (5)** | **Mixed:** Cafeterias, vending machines, and supermarkets | Pricing, combined | **Primary outcome(s):** Change in consumption of foods and beverages; data on sales/purchase were used as proxies for consumption.  **Secondary outcome(s):** Change in body weight and BMI | - Subsides (price decrease) of healthful foods significantly increases their consumption, while taxation (price increase) of unhealthful foods and beverages reduces their consumption - Each 10% decrease in price of healthful foods, but not price increases of unhealthful foods, was associated with lower BMI - Use of subsides and combined multicomponent interventions appear most effective | Centers for Disease Control and Prevention (CDC) Community Guide grading system |
| **Alston et al., 2020 (6)** | **Mixed:** Grocery stores, restaurants, farmers’ markets, community pharmacy, and community-accessible food pantries | Combined | **Primary outcome(s):** Change in food retail environment, food purchasing and dietary patterns | - All studies showed that initiatives were effective, with positive improvements in healthiness of the food environment and/or consumer behaviour | Effective Public Healthcare Panacea Project quality assessment tool |
| **Blake et al., 2019 (3)** | **Mixed:** Food service, grocery stores, cafeterias/cafés, full-service restaurants (full- and quick-service), corner stores, supermarkets, vending machines or a mix of these | Pricing, promotion, proximity, product availability, combined | **Primary outcome(s):** Commercial viability, retailer perceptions, customer perceptions, community outcomes | - Commercial viability: Favourable outcomes were found for 48% of product strategies, 19% of pricing strategies, 0% of placement strategies, and 16% of promotion strategies - Retailer perceptions: Favourable outcomes were found for 100% of product strategies, 0% of pricing and placement strategies, and 80% of promotion strategies - Customer perceptions: Favourable outcomes were found for 58% of product strategies, 100% of pricing strategies, 50% of placement strategies and 83% of promotion strategies. - Community outcomes: Favourable outcomes were found for 100% of product strategies - There was a large heterogeneity in outcome measures reported and in favourability for retailers of outcomes across studies | Mixed Methods Appraisal Tool (MMAT) |
| **Cameron et al., 2016 (8)** | Supermarkets, grocery stores, online stores (real-world or simulated) | Prompting | **Primary outcome(s):** Store sales data, self-reported food purchase data, consumer food consumption and physical measures such as BMI | - Most high quality studies targeting the supermarket food environment reported improvements in the healthiness of consumer purchases in response to the intervention. - Shelf labelling (particularly using nutrition summary scores) appears to be promising | Quality Assessment Tool for Quantitative Studies |
| **Crockett et al., 2018 (10)** | **Mixed:** Real-world settings (grocery stores, restaurants, cafeterias, coffee shop or other contexts in which food and drink for immediate consumption are sold) or laboratory settings | Prompting | **Primary outcome(s):** Food or drink purchases or consumption, and harm or unintended consequences of nutritional labels | - The results from a small body of low quality evidence suggest that nutritional labelling comprising energy information on menus may reduce energy purchased in restaurants. The evidence assessing the impact on consumption of energy information on menus or placed on a range of food options in laboratory settings suggests a similar effect to that observed for purchasing, although the evidence is less definite and of low quality | Cochrane Collaboration Risk of Bias tool |
| **Fergus et al., (13)** | **Mixed:** Tiendas, food/community stores, convenience stores, supermarkets, grocery stores, small stores, corner stores, bodegas | Prompting, proximity | **Primary outcome(s):** Nutrition-related outcomes, including customer purchases (a proxy for intake) and the intake of targeted foods (assessed via self-report, customer interview and in-store observation), and store outcomes (store environment, storeowner participation and storeowner attitudes) | - Overall, most effective interventions included point-of-purchase signage and product placement strategies. Rural studies included financial incentives combined with participant education and incorporated culturally appropriate messengers and/or symbols to improve healthy food purchases and intake. | Quality Assessment Tool for Quantitative Studies |
| **Gittelsohn et al., 2017 (14)** | **Mixed:** Grocery stores and supermarkets, all retailers in a setting (e.g., city, neighbourhood), farmer’s markets, worksite cafeteria and school cafeterias, food delivery services, carryout restaurants, corner stores, and other types of retailers | Pricing, combined | **Primary outcome(s):** Availability, purchasing or consumption of (un)healthy foods and beverages at the consumer and retail levels | - Pricing interventions, alone or combined with other strategies seemed effective in changing consumer behaviour (i.e., increased purchasing and consumption of healthy foods and beverages, and reduced purchasing and consumption of unhealthy foods and beverages). Pricing interventions appeared to have a positive impact at the retail level, with an increase in sales, stock, total profits of heathier food and beverage options. | Quality of research, ranked on a scale of 0 to 10 |
| **Golding et al., 2022 (16)** | Physical supermarket stores | Pricing, promotion, prompting | **Primary outcome(s):** Objectively measured (e.g., store sales data, shoppers’ receipts) food and non-alcoholic drink purchases | - Provision of nutritional information alone appears to be ineffective at changing shoppers’ purchasing behaviour in supermarkets. The use of price promotions in various forms of the use of prompts/cues in the environment (e.g., shelf tags) appear to be generally effective at changing purchasing behaviour across a range of products (although not all), and there is tentative evidence that drawing on the social influence of others may also be effective at changing behaviour in this context. - Effective interventions included price promotions and/or in-store merchandising. | Revised Graphic Appraisal Tool for Epidemiology (GATE) checklist for quantitative intervention studies |
| **Harbers et al., 2020 (17)** | **Mixed:** Cafeterias, supermarkets, and small food stores | Prompting, proximity | **Primary outcome(s):** Food purchases, energy intake or energy content of purchases or food choice | - Information and position nudges might be effective in improving outcomes, especially purchasing outcomes | Quality Assessment Tool for Quantitative Studies |
| **Hartmann-Boyce et al., 2018 (18)** | **Mixed:** Real supermarkets, real corner/convenience stores, and simulated (physical, web-based) | Pricing, prompting, healthy default picks, combined | **Primary outcome(s):** Purchasing behaviour (e.g., sales or transaction data) of any foods, non-alcoholic drinks, nutrients, energy, or products belonging to a defined dietary pattern or with defined dietary scores  **Secondary outcome(s):** Participants consumption of the above items | - Economic interventions showed the most promise, with 8 of the 9 studies in real store environments and all 6 studies in simulated environments detecting a statistically significant effect. The effects of these interventions appeared to be enhanced by additional promotional activity. Swap interventions appeared promising in real grocery stores, but only 2 studies tested them in this context. Interventions that altered the store environment showed mixed effects. In interventions that consisted solely of consumer education, findings were positive in simulated environments but for the most part no effect was detected in real grocery stores. | Cochrane Collaboration Risk of Bias tool |
| **Hodges et al., 2023 (19)** | Multi-channel (online and offline), online, or online “virtual or mock” grocery stores | Prompting, healthy default picks | **Primary outcome(s):** Food purchases as well as outcomes such as nutrient and diet quality of foods purchased. Food preferences indicated by consumers via a survey or other method of data collection. | - Some marketing interventions, such as nutrition labelling and food swaps, may encourage healthier food choices in the online environment. | Joanna Briggs Institute (JBI) Critical Appraisal tool |
| **Hollands et al., 2019 (20)** | **Mixed:** Field settings including shops, restaurants/cafeterias, offices, vending machines in schools, or laboratory settings | Proximity, product availability | **Primary outcome(s):** Selection (with or without purchasing) and consumption of the manipulated food, alcohol, or tobacco product(s).  **Secondary outcome(s):** Selection (with or without purchasing) and consumption of products not manipulated by the intervention | - Availability interventions: For selection outcomes, results from three studies found that exposure to fewer options resulted in a large reduction in selection of the targeted food(s) (low certainty evidence). For consumption outcomes, results from two studies found that exposure to fewer options resulted in a moderate reduction in consumption of those foods, but with considerable uncertainty (low certainty evidence). - Proximity interventions: For selection outcomes, only one study with one comparison was identified, which found that food placed farther away resulted in a moderate reduction in its selection (very low certainty evidence). For consumption outcomes, results from 12 studies found that exposure to food placed farther away resulted in a moderate reduction in its consumption (low certainty evidence). - The current evidence suggests that changing the number of available food options or altering the positioning of foods could contribute to meaningful changes in behaviour, justifying policy actions to promote such changes within food environments. However, the certainty of this evidence as assessed by GRADE is low or very low. | Revised Cochrane Collaboration Risk of Bias tool for randomised trials |
| **Karpyn et al., 2020 (21)** | **Mixed:** Supermarkets, corner stores, grocery stores, and/or convenience stores | Pricing, proximity, product availability, combined | Purchasing-related (i.e., objective store sales data, objective food purchasing data, customer receipts, and survey self-reported purchases or expenditures, store sales, or intent to purchase), and/or consumption-related (i.e., food frequency questionnaire (FFQ), 24-h dietary recall, food diary, Veggie MeterTM or other biometrics, or other survey self-reported diet/consumption or intent to eat). | - 27 out of 30 single-component interventions had at least one positive effect on improving consumptions and purchasing of healthier foods; 29 out of 34 multi-component interventions found a positive effect. Promotion strategies often had positive effects, either in combination with other approaches, or used alone. | Cochrane Collaboration Risk of Bias tool |
| **Liberato et al., 2014 (22)** | **Mixed:** Supermarkets or stores, vending machines, and an online supermarket | Pricing, prompting, combined | **Primary outcomes:** Nutritional/food intake; food purchasing.   **Secondary outcomes:** dietary biomarkers, consumer awareness, consumer knowledge, consumer self-efficacy, consumer outcome expectation, consumer beliefs, consumer attitudes, shelf-label use, shelf-label recall, healthier food stocking, availability, quality, store retail practices, policy, management and/or organisational practices, anthropometry, physiological measures, population health data such as mortality and/or morbidity data. | - There was considerable heterogeneity between the nature of the interventions, intervention duration, intensity of nutrition education activities delivered, and outcomes reported. As a result of this heterogeneity, assessments of the overall effectiveness of point-of-sale interventions in increasing purchase and/or intake of healthier food options were unable to be made - The evidence from this review indicates that monetary incentives offered to customers for a short-term seem promising in increasing purchase of healthier food options when the intervention is applied by itself in stores or supermarkets. There were insufficient studies to draw clear conclusions on the effectiveness in increasing purchase and/or intake of healthier food options when any of the interventions described in this review were applied. | Quality Assessment Tool for Quantitative Studies |
| **Mah et al., 2019 (23)** | **Mixed:** Supermarkets, grocery stores, or a combination of these, convenience stores, convenience with supermarkets, and convenience with grocery | Pricing, promotion, proximity, product availability | **Primary outcome(s):** Purchasing, dietary intake, diet quality, or diet-related health including weight (subjective or objective) | - Confirming earlier systematic reviews, the majority of interventions showed at least one positive effect on a diet-related outcome, particularly among 4P strategies. Among 4P studies, a comparable proportion of articles reporting on multicomponent strategies and single component strategies had mixed and null effects. | N/A |
| **Nikniaz et al., 2020 (24)** | **Mixed:** Schools, community settings, health care centres, supermarkets | Promotion, combined | **Primary outcome:** Increase in amount or times of dairy consumption | - Interventions reported in 18 studies were effective in increasing dairy consumption and ineffective in seven studies - It seems that applying all of the interventions together can be more effective. Interventions in health centres and supermarkets are more effective than the community interventions. - Educational interventions and changing buying/selling pattern were more effective than multiple interventions. | JBI Critical Appraisal tool for Randomised Controlled Trials and Quasi-Experimental Studies |
| **Nikolaus et al., 2016 (25)** | Grocery stores and supermarkets | Promotion | **Primary outcome(s):** Knowledge change and food-related behaviour change (e.g., purchasing, or dietary behaviour). Food-related behaviour change was assessed using either self-report or objective measures. | - The 8 studies measured additional health-related outcomes, such as participant intentions, attitudes, dietary behaviour, and purchasing behaviours. The current researchers extracted characteristics from the 6 articles that reported statistical analyses. Of these 6 studies, 5 found positive change in at least 1 health related outcome. - Constraints of included studies made it difficult to develop strong conclusions regarding characteristics that define an effective grocery store tour. - Grocery store tours are increasingly used as an avenue for nutrition education to improve knowledge and/or alter food selection behaviours and may result in positive outcomes, but it is unknown whether these outcomes persist for longer than 3 months after the tour and whether there are common attributes of effective grocery store tours. | A quality criteria checklist was used to rate each study as negative, neutral, or positive. |
| **Shangguan et al., 2019 (27)** | **Mixed:** Cafeterias, restaurants, supermarkets, shops, vending machines, and laboratories | Prompting | **Primary outcome(s):** Consumer behaviours (dietary consumption of labelled foods/beverages and sales/purchases data as proxy measures for consumption; industry responses (i.e., changes in formulations or availabilities of products); and diet related health measures, including adiposity (e.g., weight, BMI), metabolic risk factors (e.g., blood pressure, serum lipids), and clinical endpoints (e.g., coronary heart disease, diabetes mellitus) | - Food labelling reduced consumer consumption of total energy and total fat, while increasing consumption of vegetables. Food labelling did not significantly alter consumer intakes of other dietary targets including sodium, total carbohydrate, protein, saturated fat, fruits, or whole grains. | Study quality was based on five criteria: study design, assessment of exposure, assessment of outcome, control for confounding, and evidence of selection bias. |
| **Shaw et al., (28)** | **Mixed:** Supermarkets, convenience stores, petrol stores, general merchandise, liquor stores, | Combined | **Primary outcome(s):** Dietary intake, food sales data, and body composition | - The majority of studies showed that greater availability and more prominent positioning of healthy foods, or reduced availability and less prominent positioning of unhealthy foods, related to better dietary-related behaviours. A large number of results, however, were nonsignificant, which likely reflects the methodological difficulties inherent in this research field. - Both observational and intervention literature indicated moderate evidence for product positioning strategies in food stores affecting dietary-related health outcomes; most intervention studies indicated that more prominent positioning of healthy foods, and less prominent positioning of unhealthy foods, results in better dietary, or healthier sales, behaviours. - More prominent placement strategies are associated with higher sales and consumption of both healthy and unhealthy foods, but not weight status. | Predefined assessment criteria based on those described by the National Health Service (NHS) Centre for Reviews and Dissemination |
| **Slapø et al., 2021 (29)** | Real physical grocery stores and real online grocery stores | Pricing, combined | **Primary outcome:** Change in objective purchase behaviour (sales data or self-report) | - Price discounts alone or in combination with in-store promotion seem to be the most promising in terms of increasing sales of healthy food options | Cochrane Collaboration Risk of Bias tool |
| **Valencic et al., 2022 (30)** | Experimental and real-life online grocery stores, 3D virtual supermarket (web-based), or 3D virtual reality supermarket | Combined | **Primary outcome(s):** Food choices or nutrient content of food choices | - Fifteen records met the inclusion criteria, and four common nudging strategies were identified in these studies: i) applying different (already established) label(s) (n=6), ii) healthier swap suggestions (n=3), iii) default options (n=2), iv) increasing the salience (n=1) or a combination of strategies (n=3). While multiple studies identified improved nutritional contents outcomes due to nudging, overall, studies frequently lack of a clear explanation and description of the implemented visual UI features, limiting insights into which factors attracts consumer’s attention, and therefore further predicts consumer behaviours and decision making. | N/A |
| **Volkova et al., 2015 (40)** | **Mixed:** Supermarkets and restaurants (real and laboratory) | Prompting | **Primary outcome(s):** Consumer choices and purchasing behaviour (self-report or objective). | - Despite a growing body of research on POP nutrition information, real world impact of such information on consumer food choices remains contentious. There is growing evidence supporting the ability of POP information, particularly FOP nutrition labels, to enable consumers to better determine the healthiness of food products | N/A |
| **von Philipsborn et al., 2019 (33)** | **Mixed:** Schools, supermarkets, restaurants and hospitals, among others | Pricing, promotion, prompting, combined | **Primary outcome(s):** Direct or indirect measures of SSB intake, diet-related anthropometric measures and health outcomes, or any reported adverse outcome | - **Labelling interventions:** Moderate-certainty evidence that traffic-light labelling is associated with decreasing sales of SSBs, and low-certainty evidence that nutritional rating score labelling is associated with decreasing sales of SSBs. For menu-board calorie labelling reported effects on SSB sales varied - **Economic tools:** Moderate-certainty evidence that price increases on SSBs are associated with decreasing SSB sales. For price discounts on low-calorie beverages reported effects on SSB sales varied - **Whole food supply interventions:** Reported associations between voluntary industry initiatives to improve the whole food supply and SSB sales varied - **Adverse outcomes:** Negative effects on revenue, compensatory SSB consumption outside school when the availability of SSBs in schools is reduced, reduced milk intake, stakeholder discontent, and increased total energy content of grocery purchases with price discounts on low-calorie beverages, among others. The certainty of evidence on adverse outcomes was low to very low for most outcomes - **Intersectoral approaches:** Moderate-certainty evidence that government food benefit programmes with restrictions on purchasing SSBs are associated with decreased SSB intake. For unrestricted food benefit programmes reported effects varied. Moderate-certainty evidence that multicomponent community campaigns focused on SSBs are associated with decreasing SSB sales. Reported associations between trade and investment liberalisation and SSB sales varied - We found moderate-certainty evidence for consistent associations with decreases in SSB consumption or sales for the following interventions: traffic light labelling, price increases on SSB, in-store promotion of healthier beverages in supermarkets, government food benefit programs with incentives for purchasing fruits and vegetables and restrictions on SSB purchases, multi-component community campaigns focused on SSB, and interventions improving the availability of low-calorie beverages in the home environment. For the remaining interventions we found low- to very-low-certainty evidence for associations showing varying degrees of consistency. | Effective Practice and Organisation of Care (EPOC)-adapted Cochrane Collaboration Risk of Bias tool |
| **Wyse et al., 2021 (35)** | **Mixed:** Online supermarkets, online school canteens and cafeterias and online workplace cafeterias | Combined | **Primary outcome(s):** Food and beverage purchases, using sales/purchases data, or direct observation data  **Secondary outcome(s):** Unintended adverse events, economic data/evaluation | - Despite a relatively small number of studies across a range of settings, meta-analysis suggested that these interventions were effective in reducing the energy, fat, saturated fat and sodium content of online food orders - Translating information: translating existing, decision-relevant information by changing the format or presentation of the information, but not the context. - Making information visible: making external information, that is normally invisible, visible (e.g., daily calories allowances). - Providing a social reference point: role modelling, or referring to the behaviour of peer groups. - Changing choice defaults: setting no-action defaults, or the use of prompted choice (e.g., nudge). - Changing option related effort: changing the physical or financial effort to encourage or discourage certain choices. - Changing the range or composition of options: changing categories or changing the grouping of options. - Changing option consequences: changing the social consequences of certain decisions, or connecting decisions to benefits or costs (e.g., price promotions or discounts). - Providing decision assistance: providing reminders, or facilitating commitment (e.g., self or public commitment). | Cochrane Collaboration Risk of Bias tool |

*Note:* ^#^, indicates the strategy to which the interventions have been categorised, based on original framework by Kraak et al. (42) and adapted by Slapø et al. (29).

*Abbreviations:* BMI, body mass index; CDC, Centers for Disease Control and Prevention; EPOC, Effective Practice and Organisation of Care; FFQ, food frequency questionnaire; FOP, front-of-pack; GATE, Graphic Appraisal Tool for Epidemiology; JBI, Joanna Briggs Institute; N/A, not applicable; POP, point-of-purchase; PRISMA, Preferred Reporting Items for Systematic Reviews and Meta-Analyses; SSB, sugar-sweetened beverages.

**Appendix G: Characteristics of high-quality primary research studies included in the review**

Table S7. Characteristics of primary research studies

| **Reference**  **[author, year]; Study location** | **Source(s)^^^** | **Target population; Study setting^*^; Intervention duration** | **Study design** | **Treatment(s)^#^** | **Outcome(s) [Outcome measurement]** |
| --- | --- | --- | --- | --- | --- |
| Achabal et al. 1987 (43), United States | Cameron et al. (8); **Golding et al. (16)**; Hartmann-Boyce et al. (18); Liberato et al. (22); Slapø et al. (29) | Regular shoppers (n=NR); Supermarkets (n=372); 4 weeks + 4 weeks follow-up | RCT | (i) Control: no intervention  (ii) **Prompting**: via in store nutrition point-of-purchase signing – nutritional label showing “key nutrients” of the product, selection advice and calorie content and picture of food item  (iii) **Prompting**: via in store nutrition point-of-purchase signing – information label showing selection advice and picture of food item | Patronage behaviour [sales data] |
| Ball et al. 2015 (44), Australia | Adam & Jensen (4); Blake et al. (3); **Golding et al. (16)**; Hartmann-Boyce et al. (18); Slapø et al. (29); von Philipson et al. (33) | Female shoppers (n=574); Supermarkets (n=NR); 3 months + 6 months follow-up | RCT | (i) Control: no intervention  (ii) **Price**: price discounts of 20% on targeted products  (iii) **Promotion**: aiming at education customers about nutrition through 8 newsletters with recipes accompanying behaviour-change and supplementary resources (including activities such as budgeting worksheets, goal-setting and self-monitoring exercises)  (iv) **Price + Promotion** | Purchase of FV, high-calorie sugar-sweetened beverages, low-calorie carbonated diet beverages and water [sales data – electronic sales transaction data]; FV consumption (servings/day) [self-report survey] |
| Ball et al. 2016 (45), Australia | **Hartmann-Boyce et al. (18)** | Socioeconomically disadvantaged female shoppers (n=211); Supermarkets (n=NR); 6 months + 6 months follow-up | RCT | (i) Control: no intervention  (ii) **Promotion**: participants received a set of 8 educational and skill-building newsletter and behaviour change resource packages. Topics included seasonality and selection of fruit and vegetables, canned/frozen fruit and vegetable options, label reading, appropriate portion sizes, cost saving, and practical strategies for increasing fruit and vegetable consumption. Emphasis was placed on affordability, convenience, taste, and increasing self-efficacy. Supermarket tours reinforced information provided in resource packs. | Purchases of FV [sales data – electronic sales data linked to loyalty card]; FV consumption [self-report surveys] |
| Berning et al. 2010 (46), United States | **Cameron et al. (8)** | Regular shoppers (n=NR); Supermarkets (n=10); 4 weeks | Pre-post intervention | (i) Control: no intervention  (ii) **Prompting**: Seven types of shelf labels identifying healthy microwave popcorn | Sales of healthy and unhealthy microwave popcorn [sales data – scanner data] |
| Brimblecombe et al. 2017 (47), Australia | **Alston et al. (6)**; Blake et al. (3); Hartmann-Boyce et al. (18) | Remote Indigenous community (n=NR); Food stores (n=20); 24 weeks + 24 weeks follow-up | Stepped wedge RCT | (i) **Price**: 20% price discount on food and drink purchases  (ii) **Price + Promotion**: consumer education strategy to increase intake of FV and water and decrease intake of sugar-sweetened beverages | Per capita purchases (g) of FV, water, artificially sweetened soft drinks, regular soft drinks, healthy food, less healthy food [sales data] |
| Bunten et al. 2022 (48), United Kingdom | **Hodges et al. (19)** | Regular shoppers (n=NR); Online supermarket (n=1); 3 weeks | Double-blind RCT | (i) Control: no intervention  (ii) **Healthy defaults + Prompting**: centred around promoting images of healthier ‘like for like’ products on selected webpages (in-aisle banners and recipe bundles). | Healthiness of purchases [sales data]  Unintended impacts [banner clicks, purchase of standard products, overall purchase and energy (kcal) purchased] |
| Cawley et al. 2015 (49), United States | Cameron et al. (8); Harbers et al. (17); **Slapø et al. (29);** von Philipson et al. (33) | Regular shoppers (n=NR); Supermarkets (n=168); 12 weeks + 104 weeks follow-up | ITS | (i) **Prompting**: guiding stars labelling. Nutrition rating system on store shelves rating products with no-star, one-star, two-stars and three stars on 102 products | Unit sales of less nutritious foods and nutritious foods [sales data] |
| Curhan 1974 (50), United States | **Cameron et al. (8)** | Regular shoppers (n=NR); Supermarkets (n=4); 7 months | Fractional factorial RCT | (i) Any or a combination of the following:  **Price**: at least 10% lower than the prevailing “normal” price  **Promotion:** An item was considered “advertised” if it was included as one of the three produce products customarily featured by the chain in its weekly newspaper advertisement.  **Proximity:** display location quality (prime location) based on researcher observation of store traffic patterns and consultation with store personnel. Generally, separate floor tables, ends of large tables, and high-traffic positions on wall counters qualified as prime locations.  **Proximity:** display space - a space allocation of at least 200% of the space allocated to an item the period immediately preceding a test period | Sales of 16 selected fresh FV [sales data] |
| Dougherty et al. 1990 (51), United States | **Cameron et al. (8)** | Regular shoppers (n=NR); Supermarkets (n=3); 6 months | Pre-post intervention | (i) **Promotion**: Educational literature - brochures using graphics were developed for each target product (lower fat dairy foods, protein-rich food, fats and oils, convenience foods)  (ii) **Promotion**: Educational literature + videocassettes in store – 3-minute videos were shown in store, focused on different products  (iii) **Promotion**: Educational literature + videocassettes + periodic visits from a registered dietitian in store – includes taste-testing, suggestions for food swaps and providing education about food labels | Food purchasing behaviour [sales data – electronic sales data] |
| Ejlerskov et al. 2018 (52), United Kingdom | **Slapø et al. (29)** | Regular shoppers (n~30,000); Supermarkets (n=9); 12 months | ITS | (i) Control: no intervention  (ii) **Proximity**: “healthy checkout” meaning that unhealthy products such as sweets and chocolate at checkout were replaced with healthier options such as dried fruit, nuts, juices and water. | Supermarket-specific data on purchases of common checkout foods [sales data – electronic scanner] |
| Foster et al. 2014 (53), United States | Adam & Jensen (4); Cameron et al. (8); Fergus et al. (13); **Golding et al. (16)**; Harbers et al. (17); Hartmann-Boyce et al. (18); Hollands et al. (20); Liberato et al. (22); Nikniaz et al. (24); Shaw et al. (28); Slapø et al. (29); von Philipson et al. (33) | Urban, high minority, low-income neighbourhoods (n=NR); Supermarkets (n=8); 6 months | Cluster RCT | (i) Control: no intervention  (ii) **Promotion + Proximity**: promotion  included signs with the recommended product’s, shelf runners, taste-testing  and free samples of recommended products. Some products were also bundled together (only for cereal and healthy beverages). Proximity included the increased number of facings and  better placement of the recommended products | Weekly sales data for each targeted product [sales data – provided by grocers] |
| Huang et al. 2006 (54), Australia | Cameron et al. (8); Hartmann-Boyce et al. (18); Liberato et al. (22); Slapø et al. (29); **Wyse et al. (35)** | Regular shoppers (n=456); Online supermarket (n=1); ~5months | RCT | (i) Control: general non-specific advice about how to eat a diet lower in saturated fat  (ii) **Healthy Defaults**: fully automated advice that recommended specific switches from selected products higher in saturated fat to alternate similar products lower in saturated fat, giving customers the option to either retain the chosen product in the basket or swap products with an alternative lower in saturated-fat | Healthiness of food purchases (saturated fat (g/100g of food)) [sales data] |
| Levy et al. 1985 (55), United States | **Cameron et al. (8)**; Liberato et al. (22) | Regular shoppers (n=NR); Supermarkets (n=20); 2 years | Cohort, repeated measures | (i) Control stores: no intervention  (ii) **Prompting + Promotion**: shelf label intervention identifying low-cholesterol and low-fat products across 14 product categories, supported by information booklets | Total sales (ounces) across 14 categories (% market share) [sales data – electronic scanning records at checkout counters] |
| Ni Mhurchu et al. 2010 (56), New Zealand | Adam & Jensen (4); Blake et al. (3); **Golding et al. (16)**; Hartmann-Boyce et al. (18); Liberato et al. (22); Nikniaz et al. (24); Slapø et al. (29); von Philipson et al. (33) | Ethnically diverse, regular supermarket shoppers (n=254); Supermarkets (n=8); 24 weeks + 24 weeks follow-up | RCT | (i) Control: no intervention  (ii) **Promotion**: in-person nutrition education within the store. Educations recommended brand-specific healthier  alternatives to less healthy foods usually purchased  (iii) **Price**: 12.5% price reduction on eligible healthier food products  (iv) **Promotion + Price** | Healthiness of food purchases (% energy from saturated fat, energy density and % energy from macronutrients, quantities of healthier foods (by weight)) [sales data – electronic sales data] |
| Muller 1984 (57), Canada | **Cameron et al. (8)** | Regular shoppers (n=NR); Supermarkets (n=2); 2 weeks | Controlled trial | (i) **Prompting**: shelf label intervention with eight different variants of nutrition (nutrient) information for five test products at point-of-purchase | Sales of brands that are nutritionally highly ranked versus brands that are ranked lower [sales data] |
| Nikolova & Inman 2015 (58), United States | **Cameron et al. (8)** | Regular shoppers (n=535,000); Supermarkets (n=128); 6 months | Quasi-experimental field study | (i) **Prompting**: Simplified point-of-sale nutrition scoring shelf label system (NuVal) | Healthiness of food choices [sales data] |
| Paine-Andrews 1996 (59), United States | **Afshin et al. (5)**; Cameron et al. (8) | Regular shoppers (n=NR); Supermarkets (n=1); 9.5 hours | Quasi-experimental, interrupted time series | (i) **Price + Promotion:** coupons were provided to shoppers to use for selected products (40 cent price reduction =~20-25% discount; price). Demonstrators used verbal encouragement to customers to sample the lower-fat products (taste test) and educated customers that lower-fat products were better for them (promotion) | Purchases of targeted lower-fat items [researcher observations] |
| Phipps et al. 2015 (60), United States | **Hartmann-Boyce et al. (18)** | Low-income households (n=58); Fresh grocer supermarket (n=1); 8 weeks + 4 weeks tapering + 6 weeks follow-up | 4-phase prospective cohort study with RCT | (i) **Price**: provision of store vouchers after purchase (rebate of 50% of the dollar amount spent on fresh or frozen FV, reduced to 25% during a tapering phase, then eliminated) | FV (servings/wk) [sales data – point-of-sale purchase data]  FV purchasing habits [participant feedback via survey] |
| Reger et al. 2000 (61), United States | **Liberato et al. (22)** | Rural communities (n>1,300); Supermarkets (n=21); 8 weeks | Pre-post intervention | (i) **Promotion + Prompting**: participants taste-tested whole, 2%, 1%, and fat-free unflavoured milk (promotion) + store signage encouraging customers to choose low-fat milk (prompting) | Milk sales (% market share) [sales data] |
| Schucker et al. 1992 (62), United States | **Cameron et al. (8)** | Regular shoppers (n=NR); Supermarkets (n=20); 2 years | Controlled trial | (i) Control stores: no intervention  (ii) **Prompting + Promotion**: shelf label intervention identifying low-cholesterol and low-fat products across 16 product categories (prompting), supported by information booklets (promotion) | Total sales (ounces) across 16 categories (% market share) [sales data – electronic scanning records at checkout counters] |
| Silzer et al. 1994 (63), Canada | Liberato et al. (22); **Nikolaus et al. (25)** | Regular shoppers(n=124); Supermarket (n=NR); 2 hours + 1 month follow-up | Non-RCT | (i) Control: no intervention  (ii) **Promotion**: 2-hr dietitian-led supermarket nutrition education tours (focused on low fat, low sodium foods and high fibre foods; label education; food preparation) | Skills and behaviour were assessed 1-month after the intervention: frequency and ‘yes’/’no’/’do not know’ format asked types of food purchased, information read on labels, and food preparation practices |
| Smith et al. 2013 (64), New Zealand | **Hartmann-Boyce et al. (18)** | Food insecure households (n=214); Supermarkets (n=NR); 4 weeks | RCT | (i) Control: no intervention  (ii) **Price**: households received weekly food vouchers (coupons) from a supermarket (mean NZ$17/week) | Expenditure on FV, expenditure on specific food groups, total food expenditure [sales data – shopping receipts] |
| Waterlander et al. 2013 (65), The Netherlands | Adam & Jensen (4); Afshin et al. (5); Blake et al. (3); Fergus et al. (13); **Golding et al. (16)**; Hartmann-Boyce et al. (18); Liberato et al. (22); Slapø et al. (29) | Low SES regular shoppers (n=199); Supermarkets in rural areas (n=4); 6 months + 3 months follow-up | RCT | (i) Control: no intervention  (ii) **Price**: price discount of 50% for fruits and vegetables through coupons  (iii) **Promotion**: giving customers education about energy content of foods through recipes and books  (iv) **Price + Promotion** | Volume sales of FV/household (g) [sales data – supermarket cash receipts]; FV consumption [FFQ] |

*Note:* ^, bold text indicates source used to determine the quality rating; **^*^,** settings are physical (‘brick and mortar’) stores, unless otherwise specified; ^#^, bold text indicates the strategy to which the treatments have been categorised, based on original framework by Kraak et al. (42) and adapted by Slapø et al. (29).

Abbreviations: FFQ, food frequency questionnaire; FV, fruit and vegetables; g, grams; hr, hour; ITS, interrupted time series; kcal, kilocalories; n, number; NR, not reported; NZ, New Zealand; RCT, randomised controlled trial; wk, week

**Appendix H: Findings of high-quality primary studies included in the review**

Table S8. Effects of interventions on sales and consumption of target products, by strategy

| **Reference**  **[author, year]^#^** | **Healthy products included in intervention information** | | **Unhealthy / less healthy products included in intervention information** | | **Overview of findings** |
| --- | --- | --- | --- | --- | --- |
|  | **Targeted product / categories** | **Outcome effect^^^** | **Targeted product / categories** | **Outcome effect^*^** |  |
| **Single component – promotion (n=7)** | | | | | |
| Ball et al. 2015 (44) ^#^ | Fruit and vegetables (fresh, tinned, and frozen), and diet or low-calorie carbonated beverages or water | No effect | Sugar-sweetened beverages | Negative | - No effect on purchase of FV, diet or low-calorie carbonated beverages or water - Increase in purchase of sugar-sweetened beverages - Effects not maintained at 6-month follow-up |
| Ball et al. 2016 (45) | Fruit and vegetables | Mixed due to outcomes | - | - | - No significant effect on FV purchasing - Intervention group consumed more vegetables than controls, but no difference for consumption of fruit - Effect maintained at 6-month follow-up |
| Curhan 1974 (50) **^#^** | 16 selected fresh FV across 4 merchandising categories (hard fruit, cooking vegetables, salad vegetables, and soft fruit | Mixed due to outcomes | - | - | - Advertising increased sales of hard fruit and cooking vegetables - No effect for soft fruit or salad vegetables |
| Dougherty et al. 1990 (51) | Lower-fat protein-rich foods, fats and oils, dairy products, and convenience foods | Mixed due to outcomes | - | - | - Sales of only 1 product (butter-flavoured granules), increased after the intervention - No effect on other food purchases was found at any of the stores, regardless of the education method used |
| Ni Mhurchu et al. 2010 (56) ^#^ | Core foods (excluding chocolate, potato chips, sports supplements, baby foods, etc) that met Tick program criteria | No effect | Less-healthy core foods (excluding chocolate, potato chips, sports supplements, baby foods, etc) that met Tick program criteria | No effect | - No difference in products purchased, and no effect seen for any nutrients (saturated fat, total fat, protein, carbohydrate, energy density, sugars & sodium) - No effect at 24-weeks follow-up |
| Silzer et al. 1994 (63) | Sound nutrition choices that will lead to increased intake of fibre and decreased intake of fat and salt | Positive | - | - | - Intervention group reported more frequent purchasing of healthier food options (lower in fat and higher in fibre/complex carbohydrates) - Intervention group reported more frequent reading of labels and preparation of healthier food options |
| Waterlander et al. 2013 (65) ^#^ | Fruit and vegetables | No effect | - | - | - No effect for purchases of FV - No effect for consumption of FV |
| **Single component – price (n=7)** | | | | | |
| Ball et al. 2015 (44) ^#^ | Fruit and vegetables (fresh, tinned, and frozen), and diet or low-calorie carbonated beverages or water | Mixed due to outcomes | Sugar-sweetened beverages | Negative | - Increase in purchase of FV (not maintained at 6-month follow-up) - No effect on purchase of diet or low-calorie carbonated beverages or water - Increase in purchase of sugar-sweetened beverages (not at 6-month follow-up) - Effects not maintained at 6-month follow-up |
| Brimblecombe et al. 2017 (47) ^#^ | Fruit, vegetables (fresh and frozen, excluding frozen potato products), artificially  sweetened beverages and water | Positive | Sugar-sweetened  beverages | Negative | - Increase in purchase of FV - Tendency for increase in other food purchases (both healthy and less healthy) - Increase in purchase of water - Increase in purchase of sugar-sweetened beverages - At 24-weeks follow-up, decrease in purchase of less healthy food and an increase in purchase of FV - No effect for other outcomes maintained at 24-weeks follow-up |
| Curhan 1974 (50) **^#^** | 16 selected fresh FV across 4 merchandising categories (hard fruit, cooking vegetables, salad vegetables, and soft fruit | Mixed due to intervention and outcomes | - | - | - No effect on sales of hard fruit, cooking vegetables, or salad vegetables - Increase in sales of soft fruit |
| Ni Mhurchu et al. 2010 (56) ^#^ | Core foods (excluding chocolate, potato chips, sports supplements, baby foods, etc) that met Tick program criteria | Mixed due to outcomes | Less-healthy core foods (excluding chocolate, potato chips, sports supplements, baby foods, etc) that met Tick program criteria | No effect | - Increase in purchase of healthier discounted foods than those without discounts (FV, cereals and cereal products, meat and alternatives, milk and milk products) - This effect was maintained at 24-weeks follow-up (after discount removed) - No effect for purchase of non-discounted, less-healthy foods - No effect seen for any nutrients (saturated fat, total fat, protein, carbohydrate, energy density, sugars & sodium) |
| Phipps et al. 2015 (60) | Fruit and vegetables | Positive | - | - | - Intervention group purchased more servings of FV per week, and more servings of fruits and vegetables (when analysed separately) - Effects not maintained at 6-weeks follow-up - Intervention group reported they bought more FV that were more nutritious and/or bought FV that was new to them |
| Smith et al. 2013 (64) | Fruit and vegetables and specified food groups: dairy products, milks, wholegrain/wholemeal breads and meats and poultry. | Mixed due to outcomes | - | - | - Total expenditure and food expenditure was higher in intervention group - No effect for expenditure of FV, meat and poultry or dairy products - Intervention tended to spend more on potatoes and kumara and beverages |
| Waterlander et al. 2013 (65) ^#^ | Fruit and vegetables | Positive | - | - | - Increase in purchases of FV - Increase in consumption of FV - Effect not maintained at 3-month follow-up |
| **Single component – prompting (n=5)** | | | | | |
| Achabal et al. 1987 (43) | Fresh produce (broccoli, cabbage, carrots, cauliflower, kiwi fruit tomatoes) | No effect | - | - | - No effect on sales for any of the 6 types of FV - Follow-up planned but not conducted as no intervention effect |
| Berning et al. 2010 (46) | Healthy microwave popcorn | Negative | Unhealthy microwave popcorn | Negative | - Overall, sales for popcorn decreased - Demand for and sales of healthy popcorn decreased - Demand for and sales of unhealthy popcorn increased |
| Cawley et al. 2015 (49) | Nutritious (starred) foods across 102 categories | No effect | Less nutritious (unstarred) foods across 102 categories | Positive | - Total unit sales of 102 products per week decreased - No effect for sales of nutritious foods or per cent change in sales of nutritious foods - Sales of less nutritious foods decreased – largest decreases were in canned fish and meat, soda pop, bakery items, canned vegetables, cream, eggs, prepared foods, cheese, yoghurts and cookies and crackers - This trend was similar at 104-weeks follow-up |
| Muller 1984 (57) | Nutritionally highly ranked brands across 5 products: canned cream of mushroom soup (2 brands), tomato catsup (2 brands), mayonnaise (3 brands), macaroni and cheese dinner (3 brands), and bran-type breakfast cereal (7 brands) | Mixed due to outcomes |  |  | - Sales increased for 3 of 5 products (macaroni and cheese, soup and cereal), and a trend was observed for sales of mayonnaise - No effect in sales of catsup |
| Nikolova & Inman 2015 (58) | Products with NuVal scores across 8 categories: frozen pizza, tomato products, soup, salad dressing, yogurt, spaghetti sauce, granola bars, and ice cream | Positive | - | - | - Purchases of healthier product categories was higher (for all 8 categories) - Categories with the greatest increase in healthier purchasing was seen in canned soup, ice cream, granola bars |
| **Single component – proximity (n=2)** | | | | | |
| Curhan 1974 (50) **^#^** | 16 selected fresh FV across 4 merchandising categories (hard fruit, cooking vegetables, salad vegetables, and soft fruit | Mixed due to interventions and outcomes | - | - | - Location quality increased sales of hard fruit and cooking vegetables - No effect of location quality on soft fruit or salad vegetables - ‘Bonus’ display space increased sales for all categories of products |
| Ejlerskov et al. 2018 (52) | - | - | Single-serve or small packages of common, less-healthy, checkout foods: sugary confectionary, chocolate, and potato crisps | Positive | - Purchases of common, less-healthy checkout foods decreased |
| **Single component – healthy default picks (n=1)** | | | | | |
| Huang et al. 2006 (54) | - | - | Commonly purchased food items higher in saturated fat | Positive | - Saturated fat (%) in food purchased by intervention group decreased - Lower-fat dairy products were the items most frequently added to the shopping basket, after advice was provided - No effect on cost of foods purchased |
| **Multi-component – promotion + price (n=5)** | | | | | |
| Ball et al. 2015 (44) | Fruit and vegetables (fresh, tinned, and frozen), and diet or low-calorie carbonated beverages or water | Mixed due to outcomes | Sugar-sweetened  beverages | No effect | - Increase in purchase of fruit (not maintained at 6-month follow-up) - No effect on purchase of vegetables, diet or low-calorie carbonated beverages, water or sugar-sweetened beverages - Effects not maintained at 6-month follow-up |
| Brimblecombe et al. 2017 (47) | Fruit, vegetables (fresh and frozen, excluding frozen potato products), artificially  sweetened beverages and water | Mixed due to outcomes | Sugar-sweetened  beverages | No effect | - Increase in purchase of FV - No effect on purchase of other food purchases - No effect on purchase of beverages - At 24-weeks follow-up, decrease in purchase of diet soft drinks and trend for increase in purchase of vegetables - No effect for other outcomes maintained at 24-weeks follow-up |
| Ni Mhurchu et al. 2010 (56) | Core foods (excluding chocolate, potato chips, sports supplements, baby foods, etc) that met Tick program criteria | Positive | Less-healthy core foods (excluding chocolate, potato chips, sports supplements, baby foods, etc) that met Tick program criteria | No effect | - Increase in purchase of healthier discounted foods than those without discounts (FV, cereals and cereal products, meat and alternatives, milk and milk products). - This effect was maintained at 24-weeks follow-up (after discount removed) - No effect for purchase of non-discounted, less-healthy foods - No effect seen for any nutrients (saturated fat, total fat, protein, carbohydrates, energy density, sugars & sodium) |
| Paine-Andrews 1996 (59) | 3 types of lower-fat items were targeted: lower-fat milk (½% or skim), lower-fat and fat-free salad dressings, and lower-fat frozen desserts. | Promising | - | - | - Low to moderate increases for the purchase of lower-fat counterparts of milk, frozen desserts, and salad dressing (greatest increase seen for frozen desserts) |
| Waterlander et al. 2013 (65) | Fruit and vegetables | Positive | - | - | - Increase in purchases of FV - Increase in consumption of FV - Effect not maintained at 3-month follow-up |
| **Multi-component – promotion + prompting (n=3)** | | | | | |
| Levy et al. 1985 (55) | ‘Reduced’ or ‘low in’ sodium / calories / fat / cholesterol products across 14 food categories | Positive | - | - | - Sales/market share of shelf-labelled products increased in the intervention group |
| Reger et al. 2000 (61) | Lower fat, unflavoured milk | No effect | - | - | - No effect for sales of healthier milk options as a proportion of overall milk sales between intervention |
| Schucker et al. 1992 (62) | ‘Special diet’ products targeting sodium / sodium / calories / fat / cholesterol / 2:1 or greater ratio of polyunsaturated to saturated fat products across 16 food categories | Mixed due to outcomes |  |  | - Sales/market share of 8 (out of 16) shelf-labelled products increased in the intervention group |
| **Multi-component – promotion + proximity (n=1)** | | | | | |
| Foster et al. 2014 (53) | Products categorised as healthier: skim, 1% and 2% fat milk, water, cereal (2 varieties), diet soft drinks, water frozen meals - turkey dinner meal, steak, chicken nugget | Mixed due to intervention and outcomes | Products categorised as  less healthy: whole milk, regular soft drinks | No effect | - Increase in sales of skim and 1% milk (in-aisle and at checkout) and water (at checkout) - Decrease in sale of water (in-aisle display) - No effect for sales of cereal, 2% or whole milk, or diet or regular soft drinks - Increase in some healthier frozen meals (turkey dinner and chicken nugget) |
| **Multi-component – prompting + healthy default picks (n=1)** | | | | | |
| Bunten et al. 2022 (48) | Healthier swaps for spaghetti bolognese and chicken fajitas (recipe bundles); bakery (Tortillas, mini naans, mini garlic baguette and garlic baguette; in-aisle banners); baked beans, tomato ketchup, various jams, cream of tomato soup and granola (food cupboard); drinks (cola), dairy (greek style yoghurt & natural yoghurt), frozen (soft scoop ice cream) | Mixed due to outcomes | - | - | - Intervention group increased purchase of promoted healthier versions of some items in the spaghetti bolognese recipe bundle – spaghetti, sauce and cheese, but no effect for purchase of healthier mince - Intervention group increased purchase of promoted healthier versions of all items in the fajita recipe bundle – sour cream, wraps and cheese - Increase in purchase of healthier bakery and cola products - No effect for sales of items in the food cupboard, yoghurt, ice cream |
| **Multi-component – promotion + price + proximity (n=1)** | | | | | |
| Curhan 1974 (50) **^#^** | 16 selected fresh FV across 4 merchandising categories (hard fruit, cooking vegetables, salad vegetables, and soft fruit | Unclear | - | - | - Factorial design used to yield information on the effects of combinations of variables, but the results for the combination of interventions are not reported. |

*Note:* **^#^**, indicates studies have multiple intervention arms with different strategies. Effect of each strategy are reported separately, under the respective heading; ^, positive effect is considered an increase in the outcome(s); **^*^,** positive effect is considered a decrease in the outcome

Abbreviations: FV, fruit and vegetables.

**References**

1. Cochrane Training. Cochrane Handbook for Systematic Reviews of Interventions. Higgins J, Thomas J, editors2022.

2. Joanna Briggs Institute. Critical Appraisal Tools [Available from: <https://jbi.global/critical-appraisal-tools>.

3. Blake MR, Backholer K, Lancsar E, Boelsen-Robinson T, Mah C, Brimblecombe J, et al. Investigating business outcomes of healthy food retail strategies: A systematic scoping review. Obesity Reviews. 2019;20(10):1384-99.

4. Adam A, Jensen JD. What is the effectiveness of obesity related interventions at retail grocery stores and supermarkets? —a systematic review. BMC Public Health. 2016;16(1):1247.

5. Afshin A, Peñalvo JL, Del Gobbo L, Silva J, Michaelson M, O'Flaherty M, et al. The prospective impact of food pricing on improving dietary consumption: A systematic review and meta-analysis. PLoS One. 2017;12(3):e0172277.

6. Alston L, Bolton K, Reeve E, Wong Shee A, Browne J, Walker T, et al. Retail initiatives to improve the healthiness of food environments in rural, regional and remote communities. 2020.

7. An R. Effectiveness of subsidies in promoting healthy food purchases and consumption: a review of field experiments. Public Health Nutrition. 2013;16(7):1215-28.

8. Cameron AJ, Charlton E, Ngan WW, Sacks G. A Systematic Review of the Effectiveness of Supermarket-Based Interventions Involving Product, Promotion, or Place on the Healthiness of Consumer Purchases. Current Nutrition Reports. 2016;5(3):129-38.

9. Chan J, McMahon E, Brimblecombe J. Point-of-sale nutrition information interventions in food retail stores to promote healthier food purchase and intake: A systematic review. Obes Rev. 2021;22(10):e13311.

10. Crockett RA, King SE, Marteau TM, Prevost AT, Bignardi G, Roberts NW, et al. Nutritional labelling for healthier food or non-alcoholic drink purchasing and consumption. Cochrane Database Syst Rev. 2018;2(2):Cd009315.

11. Epstein LH, Jankowiak N, Nederkoorn C, Raynor HA, French SA, Finkelstein E. Experimental research on the relation between food price changes and food-purchasing patterns: a targeted review. Am J Clin Nutr. 2012;95(4):789-809.

12. Escaron AL, Meinen AM, Nitzke SA, Martinez-Donate AP. Supermarket and grocery store-based interventions to promote healthful food choices and eating practices: a systematic review. Prev Chronic Dis. 2013;10:E50.

13. Fergus L, Seals K, Holston D. Nutrition Interventions in Low-Income Rural and Urban Retail Environments: A Systematic Review. J Acad Nutr Diet. 2021;121(6):1087-114.

14. Gittelsohn J, Trude ACB, Kim H. Pricing Strategies to Encourage Availability, Purchase, and Consumption of Healthy Foods and Beverages: A Systematic Review. Prev Chronic Dis. 2017;14:E107.

15. Glanz K, Bader MDM, Iyer S. Retail Grocery Store Marketing Strategies and Obesity: An Integrative Review. American Journal of Preventive Medicine. 2012;42(5):503-12.

16. Golding SE, Bondaronek P, Bunten AK, Porter L, Maynard V, Rennie D, et al. Interventions to change purchasing behaviour in supermarkets: a systematic review and intervention content analysis. Health Psychology Review. 2022;16(2):305-45.

17. Harbers MC, Beulens JWJ, Rutters F, de Boer F, Gillebaart M, Sluijs I, et al. The effects of nudges on purchases, food choice, and energy intake or content of purchases in real-life food purchasing environments: a systematic review and evidence synthesis. Nutrition Journal. 2020;19(1):103.

18. Hartmann-Boyce J, Bianchi F, Piernas C, Payne Riches S, Frie K, Nourse R, et al. Grocery store interventions to change food purchasing behaviors: a systematic review of randomized controlled trials. Am J Clin Nutr. 2018;107(6):1004-16.

19. Hodges L, Lowery CM, Patel P, McInnis J, Zhang Q. A Systematic Review of Marketing Practices Used in Online Grocery Shopping: Implications for WIC Online Ordering. Nutrients. 2023;15(2):446.

20. Hollands GJ, Carter P, Anwer S, King SE, Jebb SA, Ogilvie D, et al. Altering the availability or proximity of food, alcohol, and tobacco products to change their selection and consumption. Cochrane Database Syst Rev. 2019;9(9):Cd012573.

21. Karpyn A, McCallops K, Wolgast H, Glanz K. Improving Consumption and Purchases of Healthier Foods in Retail Environments: A Systematic Review. Int J Environ Res Public Health. 2020;17(20).

22. Liberato SC, Bailie R, Brimblecombe J. Nutrition interventions at point-of-sale to encourage healthier food purchasing: a systematic review. BMC Public Health. 2014;14(1):919.

23. Mah CL, Luongo G, Hasdell R, Taylor NGA, Lo BK. A Systematic Review of the Effect of Retail Food Environment Interventions on Diet and Health with a Focus on the Enabling Role of Public Policies. Curr Nutr Rep. 2019;8(4):411-28.

24. Nikniaz Z, Tabrizi JS, Ghojazadeh M, Farhangi MA, Hosseini MS, Allameh M, et al. Community-based interventions to increase dairy intake in healthy populations: a systematic review. Public Health Rev. 2020;41:18.

25. Nikolaus CJ, Muzaffar H, Nickols-Richardson SM. Grocery Store (or Supermarket) Tours as an Effective Nutrition Education Medium: A Systematic Review. Journal of Nutrition Education and Behavior. 2016;48(8):544-54.e1.

26. Seymour JD, Lazarus Yaroch A, Serdula M, Blanck HM, Khan LK. Impact of nutrition environmental interventions on point-of-purchase behavior in adults: a review. Preventive Medicine. 2004;39:108-36.

27. Shangguan S, Afshin A, Shulkin M, Ma W, Marsden D, Smith J, et al. A Meta-Analysis of Food Labeling Effects on Consumer Diet Behaviors and Industry Practices. Am J Prev Med. 2019;56(2):300-14.

28. Shaw SC, Ntani G, Baird J, Vogel CA. A systematic review of the influences of food store product placement on dietary-related outcomes. Nutr Rev. 2020;78(12):1030-45.

29. Slapø H, Schjøll A, Strømgren B, Sandaker I, Lekhal S. Efficiency of In-Store Interventions to Impact Customers to Purchase Healthier Food and Beverage Products in Real-Life Grocery Stores: A Systematic Review and Meta-Analysis. Foods [Internet]. 2021; 10(5).

30. Valencic E, Beckett E, Collins C, Seljak B, Bucher T. Digital nudging in online grocery stores: A scoping review on current p ractices and gaps. Trends in Food Science & Technology. 2022;131.

31. van 't Riet J. Sales effects of product health information at points of purchase: a systematic review. Public Health Nutr. 2013;16(3):418-29.

32. Vecchio R, Cavallo C. Increasing healthy food choices through nudges: A systematic review. Food Quality and Preference. 2019;78:103714.

33. von Philipsborn P, Stratil JM, Burns J, Busert LK, Pfadenhauer LM, Polus S, et al. Environmental interventions to reduce the consumption of sugar‐sweetened beverages and their effects on health. Cochrane Database of Systematic Reviews. 2019(6).

34. Wolgast H, Halverson MM, Kennedy N, Gallard I, Karpyn A. Encouraging Healthier Food and Beverage Purchasing and Consumption: A Review of Interventions within Grocery Retail Settings. Int J Environ Res Public Health. 2022;19(23).

35. Wyse R, Jackson JK, Delaney T, Grady A, Stacey F, Wolfenden L, et al. The Effectiveness of Interventions Delivered Using Digital Food Environments to Encourage Healthy Food Choices: A Systematic Review and Meta-Analysis. Nutrients. 2021;13(7):2255.

36. Yoder AD, Proaño GV, Handu D. Retail Nutrition Programs and Outcomes: An Evidence Analysis Center Scoping Review. J Acad Nutr Diet. 2021;121(9):1866-80.e4.

37. Cohen DA, Babey SH. Contextual influences on eating behaviours: heuristic processing and dietary choices. Obes Rev. 2012;13(9):766-79.

38. Glanz K, Yaroch AL. Strategies for increasing fruit and vegetable intake in grocery stores and communities: policy, pricing, and environmental change. Prev Med. 2004;39 Suppl 2:S75-80.

39. Mayer JA, Dubbert PM, Elder JP. Promoting nutrition at the point of choice: a review. Health Educ Q. 1989;16(1):31-43.

40. Volkova E, Ni Mhurchu C. The Influence of Nutrition Labeling and Point-of-Purchase Information on Food Behaviours. Current Obesity Reports. 2015;4(1):19-29.

41. Baethge C, Goldbeck-Wood S, Mertens S. SANRA—a scale for the quality assessment of narrative review articles. Research Integrity and Peer Review. 2019;4(1):5.

42. Kraak VI, Swinburn B, Lawrence M, Harrison P. A Q methodology study of stakeholders' views about accountability for promoting healthy food environments in England through the Responsibility Deal Food Network. Food Policy. 2014;49(P1):207-18.

43. Achabal DD, McIntyre SH, Bell CH, Tucker N. The effect of nutrition POP signs on consumer attitudes and behavior. Journal of Retailing. 1987.

44. Ball K, McNaughton SA, Le HN, Gold L, Ni Mhurchu C, Abbott G, et al. Influence of price discounts and skill-building strategies on purchase and consumption of healthy food and beverages: outcomes of the Supermarket Healthy Eating for Life randomized controlled trial. Am J Clin Nutr. 2015;101(5):1055-64.

45. Ball K, McNaughton SA, Le HN, Abbott G, Stephens LD, Crawford DA. ShopSmart 4 Health: results of a randomized controlled trial of a behavioral intervention promoting fruit and vegetable consumption among socioeconomically disadvantaged women. Am J Clin Nutr. 2016;104(2):436-45.

46. Berning JP, Chouinard HH, McCluskey JJ. Do Positive Nutrition Shelf Labels Affect Consumer Behavior? Findings from a Field Experiment with Scanner Data. American Journal of Agricultural Economics. 2011;93(2):364-9.

47. Brimblecombe J, Ferguson M, Chatfield MD, Liberato SC, Gunther A, Ball K, et al. Effect of a price discount and consumer education strategy on food and beverage purchases in remote Indigenous Australia: a stepped-wedge randomised controlled trial. Lancet Public Health. 2017;2(2):e82-e95.

48. Bunten A, Shute B, Golding SE, Charlton C, Porter L, Willis Z, et al. Encouraging healthier grocery purchases online: A randomised controlled trial and lessons learned. Nutr Bull. 2022;47(2):217-29.

49. Cawley J, Sweeney MJ, Sobal J, Just DR, Kaiser HM, Schulze WD, et al. The impact of a supermarket nutrition rating system on purchases of nutritious and less nutritious foods. Public Health Nutr. 2015;18(1):8-14.

50. Curhan RC. The Effects of Merchandising and Temporary Promotional Activities on the Sales of Fresh Fruits and Vegetables in Supermarkets. Journal of Marketing Research. 1974;11:286 - 94.

51. Dougherty MF, Wittsten AB, Guarino MA. Promoting low-fat foods in the supermarket using various methods, including videocassettes. J Am Diet Assoc. 1990;90(8):1106-8.

52. Ejlerskov KT, Sharp SJ, Stead M, Adamson AJ, White M, Adams J. Supermarket policies on less-healthy food at checkouts: Natural experimental evaluation using interrupted time series analyses of purchases. PLOS Medicine. 2018;15(12):e1002712.

53. Foster GD, Karpyn A, Wojtanowski AC, Davis E, Weiss S, Brensinger C, et al. Placement and promotion strategies to increase sales of healthier products in supermarkets in low-income, ethnically diverse neighborhoods: a randomized controlled trial. Am J Clin Nutr. 2014;99(6):1359-68.

54. Huang A, Barzi F, Huxley R, Denyer G, Rohrlach B, Jayne K, et al. The effects on saturated fat purchases of providing internet shoppers with purchase- specific dietary advice: a randomised trial. PLoS Clin Trials. 2006;1(5):e22.

55. Levy AS, Mathews O, Stephenson M, Tenney JE, Schucker RE. The Impact of a Nutrition Information Program on Food Purchases. Journal of Public Policy & Marketing. 1985;4(1):1-13.

56. Ni Mhurchu C, Blakely T, Jiang Y, Eyles HC, Rodgers A. Effects of price discounts and tailored nutrition education on supermarket purchases: a randomized controlled trial. Am J Clin Nutr. 2010;91(3):736-47.

57. Muller TE. The use of nutritive composition data at the point of purchase. Journal of Nutrition Education. 1984;16(3):137-41.

58. Nikolova HD, Inman JJ. Healthy Choice: The Effect of Simplified Point-of-Sale Nutritional Information on Consumer Food Choice Behavior. Journal of Marketing Research. 2015;52(6):817-35.

59. Paine-Andrews A, Francisco VT, Fawcett SB, Johnston J, Coen S. Health marketing in the supermarket: using prompting, product sampling, and price reduction to increase customer purchases of lower-fat items. Health Mark Q. 1996;14(2):85-99.

60. Phipps EJ, Braitman LE, Stites SD, Singletary SB, Wallace SL, Hunt L, et al. Impact of a Rewards-Based Incentive Program on Promoting Fruit and Vegetable Purchases. Am J Public Health. 2015;105(1):166-72.

61. Reger B, Wootan MG, Booth-Butterfield S. A comparison of different approaches to promote community-wide dietary change. Am J Prev Med. 2000;18(4):271-5.

62. Schucker RE, Levy AS, Tenney JE, Mathews O. Nutrition shelf-labeling and consumer purchase behavior. Journal of Nutrition Education. 1992;24(2):75-81.

63. Silzer J, Sheeshka JD, Tomasik HH, Woolcott DM. An evaluation of Supermarket Safari nutrition education tours. Journal of the Canadian Dietetic Association. 1994;55:179-83.

64. Smith C, Parnell WR, Brown RC, Gray AR. Providing additional money to food-insecure households and its effect on food expenditure: a randomized controlled trial. Public Health Nutr. 2013;16(8):1507-15.

65. Waterlander WE, de Boer MR, Schuit AJ, Seidell JC, Steenhuis IH. Price discounts significantly enhance fruit and vegetable purchases when combined with nutrition education: a randomized controlled supermarket trial. Am J Clin Nutr. 2013;97(4):886-95.
